# Supplementary material for: Nucleus of the Solitary Tract Serotonin 5-HT2C Receptors Modulate Food Intake
Source: Cell Metab. 2018 Oct 2;28(4):619–630.e5. doi: 10.1016/j.cmet.2018.07.017 (PMC6371983; doi:10.1016/j.cmet.2018.07.017)
Supplement: Document S1. Figures S1 and S2 [file mmc1.pdf]

Cell Metabolism, Volume *28*

Supplemental Information

Nucleus of the Solitary Tract

Serotonin 5-HT_{2C} Receptors Modulate Food Intake

Giuseppe D'Agostino, David Lyons, Claudia Cristiano, Miriam Lettieri, Cristian Olarte-Sanchez, Luke K. Burke, Megan Greenwald-Yarnell, Celine Cansell, Barbora Doslikova, Teodora Georgescu, Pablo Blanco Martinez de Morentin, Martin G. Myers Jr., Justin J. Rochford, and Lora K. Heisler

Supplemental Figures

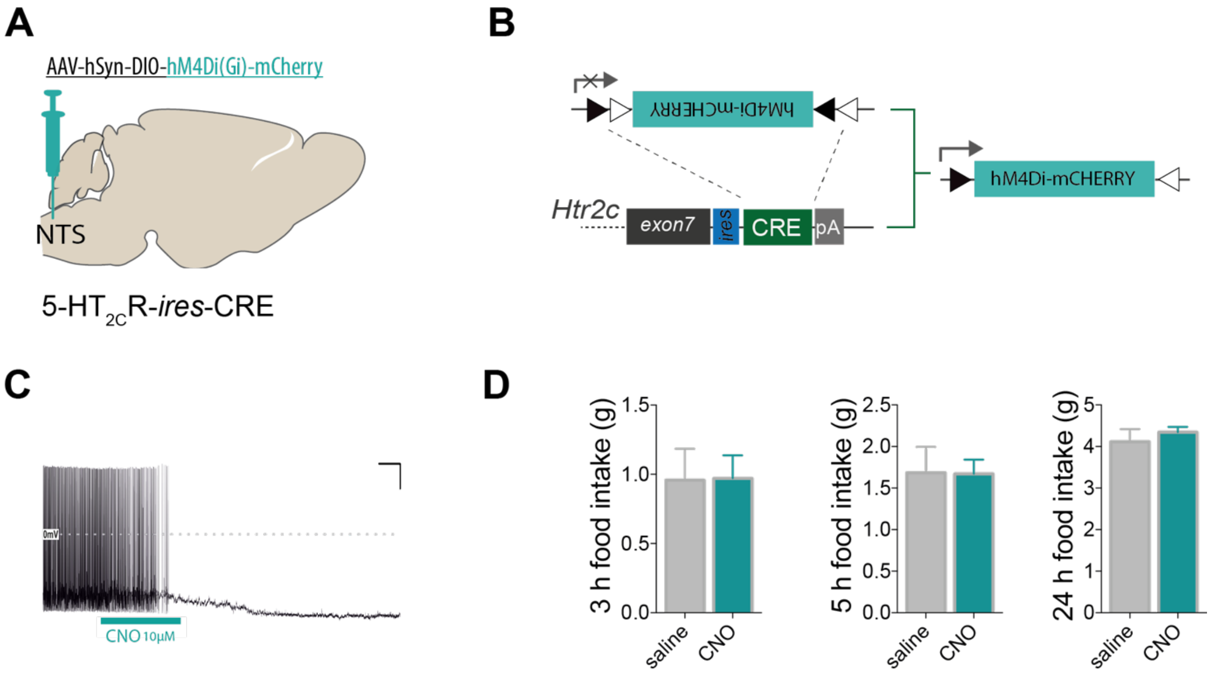

Figure S1, related to Figure 1. (A) Schematic of the strategy used to inhibit the activity of 5-HT$_{2C}$R-expressing neurons within the NTS: *5-HT$_{2C}$R^{CRE}* mice were stereotaxically injected into the NTS with a Cre-dependent DREADD vector (AAV-hM4D$_i$-mCherry). (**B**) *5-HT$_{2C}$R^{CRE}* construct and Cre-mediated recombination of DREADD allele within the NTS produced *5-HT$_{2C}$R*^{NTS}-hM4D$_i$ mice. **(C)** Representative example of electrophysiological recording of an isolated hDM4i-transduced 5-HT$_{2C}$R-expressing neuron from the NTS of *5-HT$_{2C}$R*^{NTS}-hM4D$_i$ mice illustrating that CNO (10 μM) caused a reduction in the membrane potential (n=5/5). **(D)** Chemogenetic inhibition of 5-HT$_{2C}$R^{NTS} neurons via CNO (1 mg/kg, i.p.) administration in *5-HT$_{2C}$R*^{NTS}-hM4D$_i$ mice did not alter food intake compared to saline up to 24 hours post-treatment (n= 6-7; 3h: $t(11)=0.044$, $p=0.965$; 5h: $t(11)=0.038$, $p=0.970$; 24h: $t(11)=0.642$, $p=0.533$). Data are presented as mean±SEM.

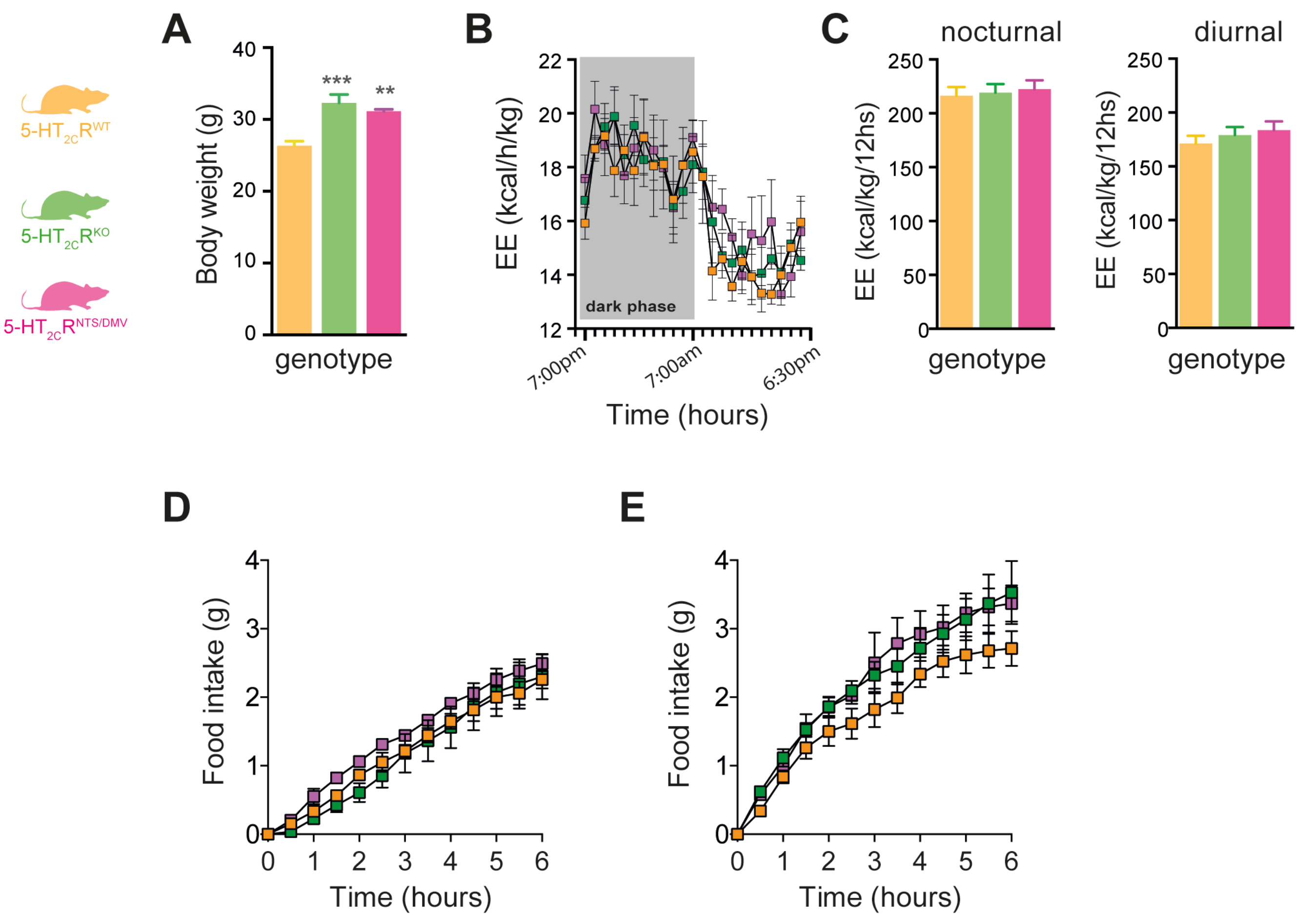

Figure S2, related to Figure 2. (A) Body weight of 4 month old male wild type (*5-$HT_{2C}R^{WT}$*), *loxtb5-$HT_{2C}R$* null mice (*5-$HT_{2C}R^{KO}$*) and *loxtb5-$HT_{2C}R$* null mice with 5-HT_{2C}Rs exclusively restored within the NTS/DMV following stereotaxic delivery of AAV-hSyn-Cre-mCherry into the NTS/DMV (*5-$HT_{2C}R^{NTS/DMV}$*) (n=5-6 per group, $F_{2,16}$= 14.40, p=0.0003). **(B)** Representative recording of circadian energy expenditure (EE) measured by indirect calorimetry illustrating no effect of genotype on EE (kcal/h/kg) during the dark or light phase of the daily cycle in *5-$HT_{2C}R^{WT}$* (n=5), *5-$HT_{2C}R^{KO}$* (n=5) or *5-$HT_{2C}R^{NTS/DMV}$* (n=6) mice **(**Effect of genotype $F_{2,13}$ = 0.26, p = 0.7739; effect of time $F_{23,299}$ = 15.56, p <0.001; interaction $F_{46,299}$ = 0.66, p = 0.9544); **(C)** Data expressed as cumulative nocturnal and diurnal EE also illustrate no differences by genotype in EE. **(D)** Cumulative *ad libitum* dark cycle food intake illustrating no differences by genotype. **(E)** Cumulative light cycle food intake after an overnight fast illustrating no significant differences by genotype. Sidak's post hoc comparisons **p < 0.01, ***p < 0.001 compared to *5-$HT_{2C}R^{WT}$* mice. Data are presented as mean±SEM.
