# Supplementary material for: Nucleus of the Solitary Tract Serotonin 5-HT2C Receptors Modulate Food Intake
Source: Cell Metab. 2018 Oct 2;28(4):619–630.e5. doi: 10.1016/j.cmet.2018.07.017 (PMC6371983; doi:10.1016/j.cmet.2018.07.017)
Supplement: Document S2. Article plus Supplemental Information [file mmc2.pdf]

Cell Metabolism

Article

Nucleus of the Solitary Tract Serotonin 5-HT_{2C} Receptors Modulate Food Intake

Graphical Abstract

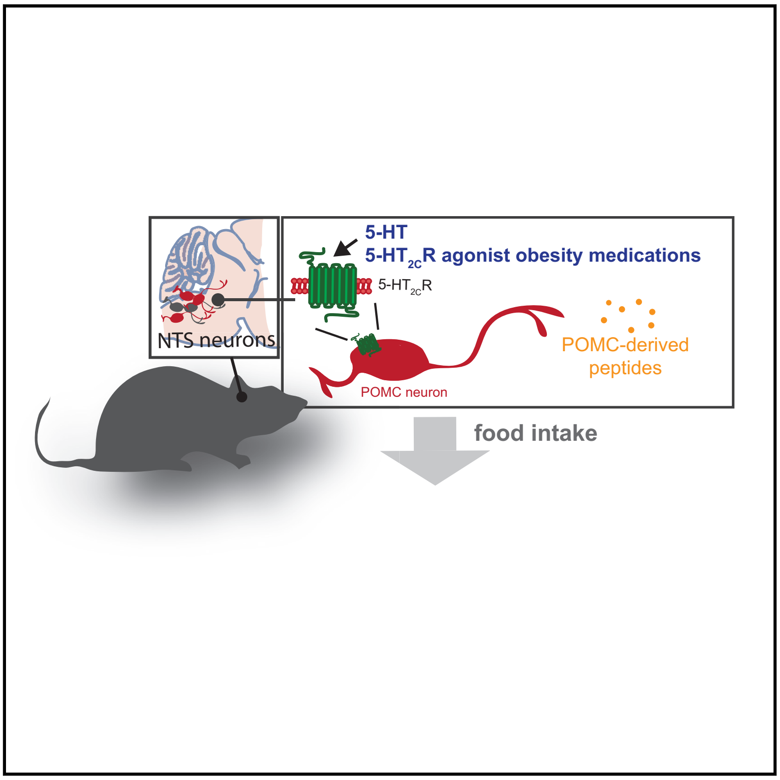

Highlights

- Selective 5-$HT_{2C}R^{NTS}$ neuron activation significantly reduces food intake
- 5-$HT_{2C}R^{NTS}$ are sufficient for obesity medication acute appetite suppression
- 5-$HT_{2C}R^{NTS}$ are co-expressed with $POMC^{NTS}$; lorcaserin activates $POMC^{NTS}$ cells
- $POMC^{NTS}$ and $POMC^{ARC}$ are required for 5-$HT_{2C}R$ agonists to reduce feeding

Authors

Giuseppe D'Agostino, David Lyons, Claudia Cristiano, ..., Martin G. Myers, Jr., Justin J. Rochford, Lora K. Heisler

Correspondence

giuseppe.dagostino@abdn.ac.uk (G.D.), lora.heisler@abdn.ac.uk (L.K.H.)

In Brief

The brain serotonin pathway regulates appetite and body weight. D'Agostino, Heisler et al. show that the anti-obesity 5-$HT_{2C}R$ agonist lorcaserin targets both the POMC circuitry of the hypothalamus and the brainstem nucleus of the solitary tract (5-$HT_{2C}R^{NTS}$) to mediate appetite suppression.

D'Agostino et al., 2018, Cell Metabolism *28*, 619–630
October 2, 2018 © 2018 The Authors. Published by Elsevier Inc.
https://doi.org/10.1016/j.cmet.2018.07.017

Nucleus of the Solitary Tract Serotonin 5-HT$_{2C}$ Receptors Modulate Food Intake

Giuseppe D'Agostino,[1,2,4,*] David Lyons,[1,4] Claudia Cristiano,[1] Miriam Lettieri,[1] Cristian Olarte-Sanchez,[1] Luke K. Burke,[2] Megan Greenwald-Yarnell,[3] Celine Cansell,[1] Barbora Doslikova,[2] Teodora Georgescu,[1] Pablo Blanco Martinez de Morentin,[1] Martin G. Myers, Jr.,[3] Justin J. Rochford,[1] and Lora K. Heisler[1,2,5,*]
[1]Rowett Institute, University of Aberdeen, Aberdeen, UK
[2]Department of Pharmacology, University of Cambridge, Cambridge, UK
[3]Division of Metabolism, Endocrinology, and Diabetes, Department of Internal Medicine, University of Michigan, Ann Arbor, MI 48109, USA
[4]These authors contributed equally
[5]Lead Contact
*Correspondence: giuseppe.dagostino@abdn.ac.uk (G.D.), lora.heisler@abdn.ac.uk (L.K.H.)
https://doi.org/10.1016/j.cmet.2018.07.017

SUMMARY

To meet the challenge to human health posed by obesity, a better understanding of the regulation of feeding is essential. Medications targeting 5-hydroxytryptamine (5-HT; serotonin) 2C receptors (*htr2c*; 5-HT$_{2C}$R) improve obesity. Here we probed the functional significance of 5-HT$_{2C}$Rs specifically within the brainstem nucleus of the solitary tract (5-HT$_{2C}$R^{NTS}) in feeding behavior. Selective activation of 5-HT$_{2C}$R^{NTS} decreased feeding and was sufficient to mediate acute food intake reductions elicited by the 5-HT$_{2C}$R agonist obesity medication lorcaserin. Similar to pro-opiomelanocortin neurons expressed within the hypothalamic arcuate nucleus (POMC^{ARC}), a subset of POMC^{NTS} neurons co-expressed 5-HT$_{2C}$Rs and were activated by 5-HT$_{2C}$R agonists. Knockdown of POMC^{NTS} prevented the acute appetite-suppressive effect of lorcaserin, whereas POMC^{ARC} knockdown prevented the full anorectic effect. These data identify 5-HT$_{2C}$R^{NTS} as a sufficient subpopulation of 5-HT$_{2C}$Rs in reducing food intake when activated and reveal that 5-HT$_{2C}$R agonist obesity medications require POMC within the NTS and ARC to reduce food intake.

INTRODUCTION

As demonstrated 40 years ago, brain 5-hydroxytryptamine (5-HT; serotonin) is a significant regulator of appetite and body weight (Breisch et al., 1976). Targeting this neurochemical machinery, medications increasing 5-HT bioavailability were developed to treat obesity. However, increasing 5-HT activity at peripheral 5-HT receptors (5-HTRs) contributed to side effects that led to the withdrawal of medications such as d-fenfluramine in the 1990s and sibutramine in the 2000s (Burke and Heisler, 2015). Efforts to delineate 5-HTRs mediating 5-HT's therapeutic effects on food intake revealed the 2C receptor subtype (*Htr2cr*, 5-HT$_{2C}$R) as the principal mediator (Tecott et al., 1995; Williams et al., 2011). The clinical significance of this discovery is underscored by a new obesity medication, the 5-HT$_{2C}$R agonist, lorcaserin, which was recently launched in the United States (Aronne et al., 2014; Smith et al., 2010).

5-HT$_{2C}$Rs are widely distributed within the brain where they act to modulate a diverse array of behaviors and physiological processes (Burke and Heisler, 2015; Julius et al., 1988). Of the neurochemically defined cells expressing 5-HT$_{2C}$Rs, it is a subpopulation of neurons within the arcuate nucleus of the hypothalamus (ARC) that co-express the precursor polypeptide pro-opiomelanocortin (POMC) that have been proposed to be a principal mediator of 5-HT$_{2C}$R's effects on metabolic functions (Berglund et al., 2013; Burke et al., 2017; Doslikova et al., 2013; Heisler et al., 2002; Xu et al., 2010). However, anorectic doses of 5-HT$_{2C}$R agonists have been shown to increase markers of neuronal activity in caudal brainstem regions, including the nucleus of the solitary tract (NTS) (Lam et al., 2009; Stark et al., 2006). Although the NTS is a brainstem structure known to be a key regulatory center for the integration of food related signals of both peripheral and central origin (Fan et al., 2004; Grill and Hayes, 2009; Zhan et al., 2013), no direct functional assessment of 5-HT$_{2C}$Rs in this brain region has been undertaken. Here we probed the functional significance of 5-HT$_{2C}$Rs within the NTS, their contribution to the therapeutic effect of 5-HT$_{2C}$R agonist obesity medications, and the mechanism underpinning their appetite-reducing effect via exclusive action within the NTS.

RESULTS

Selective 5-HT$_{2C}$R^{NTS} Neuron Activation Decreases Food Intake

To determine whether 5-HT$_{2C}$R-expressing NTS (5-HT$_{2C}$R^{NTS}) neurons play a role in appetite control, we first employed the chemogenetic designer receptors exclusively activated by designer drugs (DREADDs; Armbruster et al., 2007) approach to specifically express G$_q$-coupled (hM3D$_q$) or G$_i$-coupled (hM4D$_i$) designer receptors in 5-HT$_{2C}$R^{NTS} neurons. To target 5-HT$_{2C}$R^{NTS} neurons, we stereotactically delivered Cre-dependent adeno-associated viruses (AAVs) encoding designer receptor hM3D$_q$ or hM4D$_i$ into the NTS of *5-HT$_{2C}$R^{Cre}* mice (Burke et al., 2016, 2017; Marcinkiewcz et al., 2016) to generate *5-HT$_{2C}$R^{NTS}*-hM3D$_q$ (Figures 1A and 1B) and *5-HT$_{2C}$R^{NTS}*-hM4D$_i$ cohorts (Figures S1A and S1B). Cre recombinase activity

 © 2018 The Authors. Published by Elsevier Inc.
This is an open access article under the CC BY license (http://creativecommons.org/licenses/by/4.0/).

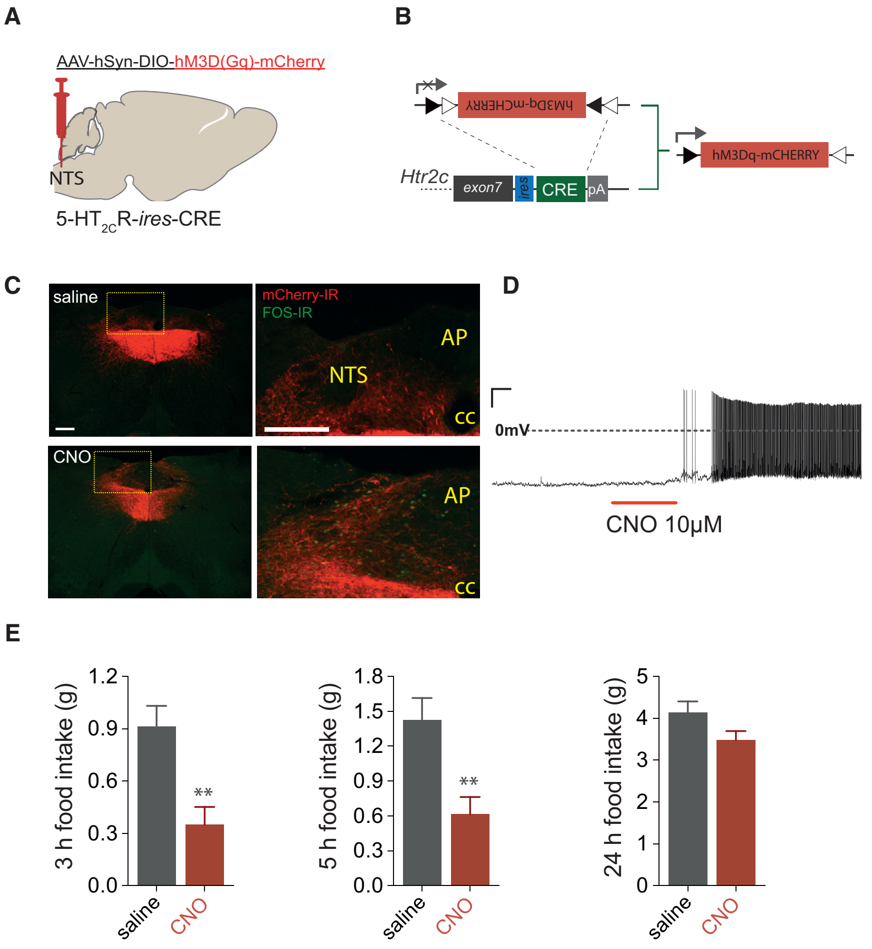

Figure 1. Activation of $5\text{-}HT_{2C}R^{NTS}$-Expressing Neurons Reduces Food Intake

(A) Schematic of the strategy used to selectively activate $5\text{-}HT_{2C}R^{NTS}$ neurons; stereotactic injection of Cre-dependent DREADD vectors into NTS of *$5\text{-}HT_{2C}R^{Cre}$* mice.

(B) *$5\text{-}HT_{2C}R^{Cre}$* construct and Cre-mediated recombination of DREADD allele.

(C) Expression of the DREADD-fused mCherry reporter protein within the NTS using immunohistochemistry for mCherry (mCherry-IR; red). *In vivo*, CNO (1 mg/kg, intraperitoneally [i.p.]) increased FOS-IR (green) expression in the majority of *$5\text{-}HT_{2C}R^{NTS}$*-hM3D_q-expressing neurons (red, bottom panels), whereas saline treatment did not (top panels).

(D) *Ex vivo*, bath application of CNO (10 μM) increased the firing frequency of *$5\text{-}HT_{2C}R^{NTS}$*-hM3D_q-expressing neurons.

(E) CNO (1 mg/kg, i.p.) elicited a significant reduction in *ad libitum* dark cycle food intake compared with saline treatment in *$5\text{-}HT_{2C}R^{NTS}$*-hM3D_q mice (n = 6–7; 3 hr: $t_{12} = 3.57$, $p = 0.004$; 5 hr: $t_{12} = 3.379$, $p = 0.005$; 24 hr: $t_{12} = 1.876$, $p = 0.0874$). Data are presented as mean ± SEM. **$p < 0.01$.

NTS, nucleus of the solitary tract; AP, area postrema; CC, central canal. Scale bar, 200 μm. See also Figure S1.

in *$5\text{-}HT_{2C}R^{Cre}$* mice efficiently recombined the DREADD-mCherry allele in the NTS, as seen by the expression of the mCherry reporter protein in transduced cells (Figure 1C). A degree of spread was observed in adjacent regions following amplification of mCherry signal with immunohistochemistry (Figure 1C). Following treatment with designer drug clozapine-N-oxide (CNO; 1 mg/kg, intraperitoneally [i.p.]), c-fos immunoreactivity (FOS-IR; a surrogate marker of neuronal activation) was observed within the NTS of *$5\text{-}HT_{2C}R^{NTS}$*-hM3D_q mice. In *$5\text{-}HT_{2C}R^{NTS}$*-hM3D_q mice that had been fasted overnight to decrease baseline FOS-IR, quantification analysis revealed that approximately 76.4% ± 3.7% of mCherry-positive NTS cells expressed FOS-IR following CNO treatment whereas less than 1% expressed FOS-IR following saline treatment (Figure 1C). Consistent with these *in vivo* data, in *ex vivo* NTS slices, bath application of CNO (10 μM) increased the firing frequency of *$5\text{-}HT_{2C}R^{NTS}$*-hM3D_q-expressing neurons (n = 5/5; Figure 1D). These data indicate that CNO allows remote chemical control of $5\text{-}HT_{2C}R^{NTS}$ neuronal activity in *$5\text{-}HT_{2C}R^{NTS}$*-hM3D_q-expressing mice. We next examined the effect of CNO on feeding behavior. CNO (1 mg/kg, i.p.) significantly reduced the first 5 hr of *ad libitum* dark cycle chow intake compared with saline treatment in *$5\text{-}HT_{2C}R^{NTS}$*-hM3D_q mice (Figure 1E).

Despite the CNO (10 μM)-induced reduction in membrane potential observed in *ex vivo* NTS slices from *$5\text{-}HT_{2C}R^{NTS}$*-hM4D_i mice (n = 5/5; Figure S1C), CNO (1 mg/kg, i.p.) did not affect *ad libitum* food intake in *$5\text{-}HT_{2C}R^{NTS}$*-hM4D_i mice compared with saline (Figure S1D). The effect of CNO on feeding behavior in *$5\text{-}HT_{2C}R^{NTS}$*-hM3D_q but not *$5\text{-}HT_{2C}R^{NTS}$*-hM4D_i mice illustrates that CNO is not altering food intake via action at endogenous receptors. Rather, the designer drug CNO is reducing food intake via action at the designer *$5\text{-}HT_{2C}R^{NTS}$*-hM3D_q receptors. These data reveal that activation of $5\text{-}HT_{2C}R^{NTS}$ neurons significantly suppresses feeding and identifies a novel population of NTS cells that may be targeted for food intake reduction.

Selective $5\text{-}HT_{2C}R^{NTS/DMV}$ Activation Is Sufficient to Promote $5\text{-}HT_{2C}R$ Agonist Hypophagia

We next evaluated whether an obesity medication currently in human use employs the subset of $5\text{-}HT_{2C}R^{NTS}$ to promote a reduction in feeding behavior. To exclusively activate $5\text{-}HT_{2C}R^{NTS}$ with clinical and preclinical obesity medications, we restricted the expression of the endogenous receptor to the caudal aspects of the dorsal vagal complex (the NTS and dorsal motor nucleus of the vagus [DMV]; *$5\text{-}HT_{2C}R^{NTS/DMV}$*). We achieved this using a reversible *$5\text{-}HT_{2C}R$* null line, in which expression of *Htr2cr* is prevented by a loxP-flanked transcriptional blocker (Xu et al., 2008). The expression of *Htr2cr* was site-specifically reactivated via stereotactic delivery of an AAV-expressing Cre recombinase (AAV-Cre) into the NTS/DMV, which removes the transcriptional blocker (Figures 2A and 2B), allowing $5\text{-}HT_{2C}Rs$ expression only in the NTS/DMV (Figures 2C and 2D). To confirm the functional re-expression of *Htr2c*,

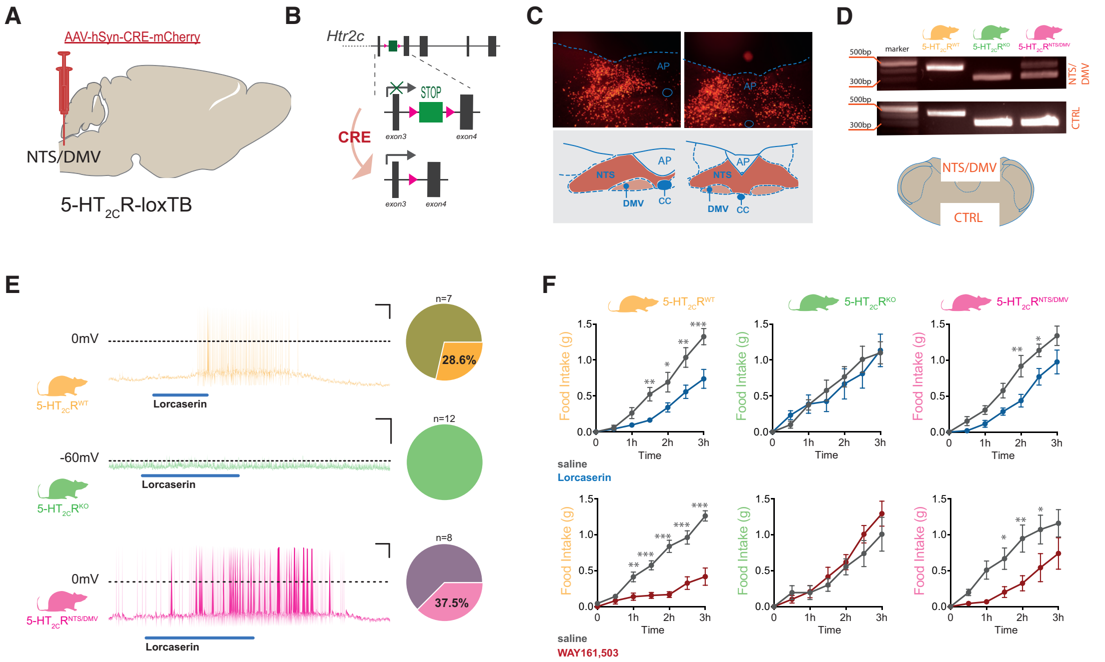

Figure 2. 5-HT$_{2C}$R^{NTS} Is Sufficient to Mediate the Appetite-Suppressive Effect of 5-HT$_{2C}$R Agonists

(A) Schematic of the strategy used to selectively restore 5-HT$_{2C}$Rs within the NTS of *loxTB5-HT$_{2C}$R* null mice through stereotactic injection of Cre-dependent DREADD vectors into NTS.

(B) *5-HT$_{2C}$R* allele expression is disrupted by a loxP-flanked transcriptional blocker (loxTB) inserted between exons 3 and 4 of the *htr2c* gene in *loxTB5-HT$_{2C}$R* mice (*5-HT$_{2C}$R^{KO}*). Stereotactic injection of AAV vector expressing Cre recombinase and mCherry reporter driven by the hSyn promoter removes the TB and allows 5-HT$_{2C}$R expression only in the NTS (*5-HT$_{2C}$R$^{NTS/DMV}$*).

(C) Representative image of Cre-mCherry expression within the rostrocaudal aspect of the NTS/DMV.

(D) Cre-mediated recombination of the mutant allele and TB removal specifically within the NTS/DMV in *5-HT$_{2C}$R$^{NTS/DMV}$* mice (wild-type band 437 bp, mutant 310 bp).

(E) Representative current-clamp traces and proportion of neurons responding to bath application of lorcaserin in *ex vivo* NTS slices prepared from *5-HT$_{2C}$R^{WT}* (n = 7), *5-HT$_{2C}$R^{KO}* (n = 12), and *5-HT$_{2C}$R$^{NTS/DMV}$* (n = 8) mice.

(F) Sidak's post hoc comparisons revealed that lorcaserin and WAY161,503 (7 mg/kg, i.p.) reduced *ad libitum* food intake at specific time points within the first 3 hr of the dark cycle (tick marks on x axis represent 20-min intervals) in *5-HT$_{2C}$R^{WT}* (lorcaserin, n = 5; time: $F_{6,24} = 55.19$, $p < 0.0001$; treatment: $F_{1,4} = 16.81$, $p = 0.0149$; interaction: $F_{6,24} = 8.991$, $p < 0.0001$. WAY161,503, n = 6; time: $F_{6,30} = 53.37$, $p < 0.0001$; treatment: $F_{1,5} = 61.55$, $p = 0.0005$; interaction: $F_{6,30} = 26.16$, $p < 0.0001$) and *5-HT$_{2C}$R$^{NTS/DMV}$* mice (lorcaserin, n = 5; time: $F_{6,24} = 63.53$, $p < 0.0001$; treatment: $F_{1,4} = 23.78$, $p = 0.0082$; interaction: $F_{6,24} = 4.099$, $p = 0.0057$. WAY161,503, n = 6; time: $F_{6,30} = 51.89$, $p < 0.0001$; treatment: $F_{1,5} = 4.028$, $p = 0.1010$; interaction: $F_{6,30} = 1.878$, $p = 0.1175$), but not *5-HT$_{2C}$R^{KO}* mice. Data are presented as mean ± SEM. Sidak's post hoc comparisons *$p < 0.05$, **$p < 0.01$, ***$p < 0.001$.

NTS, nucleus of the solitary tract; AP, area postrema; CC, central canal; DMV, dorsal motor nucleus of the vagus. See also Figure S2.

we made *ex vivo* whole-cell electrophysiological recordings from neurons within NTS slices in *5-HT$_{2C}$R^{WT}* (wild-type), *5-HT$_{2C}$R^{KO}* (knockout), and mice with 5-HT$_{2C}$Rs restored exclusively within the NTS/DMV (*5-HT$_{2C}$R$^{NTS/DMV}$*). As expected, NTS neurons from *5-HT$_{2C}$R^{KO}* mice failed to respond to lorcaserin, while similar proportions of NTS neurons from *5-HT$_{2C}$R^{WT}* and *5-HT$_{2C}$R$^{NTS/DMV}$* mice were excited by lorcaserin (Figure 2E). These data indicate that AAV-Cre restores functional 5-HT$_{2C}$R expression in *5-HT$_{2C}$R$^{NTS/DMV}$* mice.

Consistent with previous reports, body weight was significantly higher in adult *5-HT$_{2C}$R^{KO}* compared with *5-HT$_{2C}$R^{WT}* mice (Nonogaki et al., 2003; Xu et al., 2008); an effect that failed to normalize following post-developmental re-expression of the 5-HT$_{2C}$R exclusively within the NTS/DMV (Figure S2A). Further analysis of energy balance revealed no difference in daily, nocturnal, or diurnal energy expenditure between genotypes (Figures S2B and S2C). We were unable to detect the predicted increase in *ad libitum* chow intake (Figure S2D) or chow intake following an overnight fast in *5-HT$_{2C}$R^{KO}* mice (Figure S2E), which may be related to the previously reported variability in the magnitude of hyperphagia on a chow diet with age in *5-HT$_{2C}$R^{KO}* mice (Wade et al., 2008). These results indicate that restoration of 5-HT$_{2C}$Rs only within the NTS/DMV is not sufficient to correct the increased body weight phenotype of chow-fed *5-HT$_{2C}$R^{KO}* mice.

To test the significance of *5-HT$_{2C}$R$^{NTS/DMV}$* in a therapeutic context, we treated *5-HT$_{2C}$R^{WT}*, *5-HT$_{2C}$R^{KO}*, and *5-HT$_{2C}$R$^{NTS/DMV}$* mice with 5-HT$_{2C}$R agonists lorcaserin and WAY161,503. In line with previous research (Burke et al., 2014; Fletcher et al., 2009), the obesity medication lorcaserin and the preclinical compound WAY161,503 significantly reduced *ad libitum* food intake in *5-HT$_{2C}$R^{WT}* mice compared with saline treatment (Figure 2F). This effect was absent in *5-HT$_{2C}$R^{KO}* mice but predominantly restored in *5-HT$_{2C}$R$^{NTS/DMV}$* mice (Figure 2F). Thus, consistent with chemogenetic activation of 5-HT$_{2C}$R^{NTS} cells, pharmacological activation of this discrete subset of cells

has a significant impact on feeding behavior. These data also reveal that 5-HT$_{2C}$Rs expressed within the NTS/DMV are sufficient to mediate acute therapeutic effects of a medication currently in clinical use to treat obesity, thereby highlighting a specific subset of 5-HT$_{2C}$Rs that may be exploited for future obesity medication development.

5-HT$_{2C}$R Agonists Directly Activate POMC^{NTS} Cells

We next interrogated the neurochemical phenotype of 5-HT$_{2C}$R^{NTS} cells to probe the mechanism through which 5-HT$_{2C}$R^{NTS}-mediated effects on appetite are achieved. Though POMC neurons within the ARC have been proposed to be a primary mediator of 5-HT$_{2C}$R's effects on metabolic functions (Berglund et al., 2013; Burke et al., 2014, 2017; Doslikova et al., 2013; Heisler et al., 2002; Xu et al., 2010), a smaller and less well characterized population of POMC expressing neurons reside within the caudal aspect of the NTS (POMC^{NTS}), a region receiving inputs from vagal afferent fibers that transmit meal-related information from the gastrointestinal system (Appleyard et al., 2005; Fan et al., 2004; Joseph et al., 1983; Zhan et al., 2013). We thus hypothesized that POMC^{NTS} is a functional component of 5-HT$_{2C}$R^{NTS}-mediated hypophagia.

To visualize anatomical expression, we utilized *Pomc*^{DsRED} reporter mice (Hentges et al., 2009) and fluorescent *in situ* hybridization to label endogenous *5-HT$_{2C}$R* mRNA. Although more than one-third (38.2% ± 1.3%) of POMC^{NTS} cells express *5-HT$_{2C}$R* mRNA, POMC^{NTS} neurons represent only a small (approximately 4%) subset of the total NTS *5-HT$_{2C}$R* mRNA-expressing population (Figure 3A). Next, to evaluate the impact of 5-HT$_{2C}$R activation on POMC^{NTS} neuronal activity, we performed whole-cell electrophysiological recordings in *Pomc*^{DsRED} NTS slices. Consistent with the anatomical co-expression profile, 45.5% (10/22) of *Pomc*^{DsRED} cells were responsive to 5-HT (10 μM), the endogenous 5-HT$_{2C}$R agonist (Figures 3B and 3C). A similar proportion of responders was observed following application of 5-HT$_{2C}$R agonists lorcaserin (20 μM) and WAY161,503 (20 μM), with 47.1% (8/17) and 50% (11/22) of *Pomc*^{DsRED} cells responding, respectively (Figures 3B and 3C). All responses were characterized by depolarization and commencement/enhancement of action potential discharge. These effects were independent of action potential-dependent synaptic transmission, as depolarizations endured in the presence of 500 nM tetrodotoxin (TTX) (5-HT, 13.2 ± 3.8 mV, n = 7; lorcaserin, 11.7 ± 3.6 mV, n = 7; WAY161,503, 12.8 ± 2.7 mV, n = 8; Figure 3C), indicating that 5-HT and 5-HT$_{2C}$R agonists directly activate POMC^{NTS} cells via a post-synaptic mechanism. 5-HT and 5-HT$_{2C}$R agonists induced a similar degree of depolarization and activated a similar percentage of POMC^{NTS} cells (Figure 3D). These data reveal that 5-HT$_{2C}$Rs are anatomically positioned to increase the activity of a subset of POMC^{NTS} cells.

To examine the post-synaptic ionic mechanisms underpinning the excitatory effects of 5-HT and lorcaserin, we recorded POMC^{NTS} neurons in a voltage clamp (VC) and in the presence of TTX. At a holding potential of −60 mV, application of 5-HT (10 μM) induced a reversible inward current of −23.1 ± 2.5 pA (n = 10; Figure 4A). The current-voltage relationship of $I_{5\text{-HT}}$ was examined using VC ramps, which drove the membrane potential from −120 mV to 0 mV at a rate of 45.7 mV s^{-1} (Figure 4B; n = 10). The digital subtraction of the ramp in control from the ramp at the peak of the response revealed $I_{5\text{-HT}}$ to be outwardly rectifying and to reverse at −27.0 ± 3.6 mV (Figure 4C; n = 10). Likewise, lorcaserin (20 μM) induced an inward current of −15.5 ± 2.5 pA (n = 12; Figure 4D) that was shown to be outwardly rectifying and to reverse at −24.6 ± 2.9 mV (Figures 4E and 4F). These data illustrate that 5-HT and lorcaserin excite POMC^{NTS} neurons via the activation of a post-synaptic, mixed cationic current. This mechanism is consistent with that observed for 5-HT$_{2C}$R activation of POMC^{ARC} neurons (Sohn et al., 2011).

Brain POMC Is Necessary to Mediate 5-HT$_{2C}$R Agonist Feeding Effects

Thus far, we have established that the 5-HT$_{2C}$Rs are anatomically positioned to influence the activity of approximately 40% of POMC^{NTS} neurons and previous reports indicate that 5-HT$_{2C}$Rs are co-expressed with approximately 40% of POMC^{ARC} neurons (Heisler et al., 2002; Lam et al., 2008). Earlier transgenic approaches have revealed that the subset of 5-HT$_{2C}$Rs that are necessary and sufficient for 5-HT$_{2C}$R agonists to reduce food intake are co-expressed with POMC (Berglund et al., 2013; Xu et al., 2008). These approaches manipulate the expression of the receptor, not POMC production. We aimed to determine the function of POMC in lorcaserin's anorectic effect. As a first step toward this goal, we employed a mouse model with full POMC^{ARC} knockout and approximately half POMC^{NTS} knockdown (*Pomc^{NEO}*) (Bumaschny et al., 2012). *Pomc^{NEO}* and wild-type (*Pomc^{WT}*) littermates were treated with saline or lorcaserin (7 mg/kg, i.p.) at the onset of the dark cycle and *ad libitum* home cage chow intake was measured 1, 3, and 6 hr later. As expected, *Pomc^{WT}* mice treated with lorcaserin significantly reduced food intake compared with saline-treated littermates at each time point (Figure 5A). However, lorcaserin did not have a significant effect on food intake at any of these time points in *Pomc^{NEO}* mice compared with saline-treated littermates (Figure 5B). These results were replicated in further cohorts of *Pomc^{NEO}* and *Pomc^{WT}* littermates using a within-subjects experimental design (data not shown). These data reveal that brain POMC is required for lorcaserin to promote its effects on food intake.

POMC^{ARC} Is Required for the Full Effect of 5-HT$_{2C}$R Agonist on Food Intake

We next sought to determine the functional significance of the discrete subset of POMC^{NTS} versus POMC^{ARC} in 5-HT$_{2C}$R agonist-induced reduction of food intake. We first targeted *Pomc* in the hypothalamus, since this population of *Pomc* expressing neurons is larger, better characterized, and projects widely within the hypothalamus, allowing efficient tissue sampling and recovery of peptides that localize at neuron terminals (e.g., POMC-derived peptide α-melanocyte-stimulating hormone [α-MSH] [D'Agostino and Diano, 2010]). To knock down POMC^{ARC}, we employed the clustered, regularly interspaced, short palindromic repeats (CRISPR) and associated endonuclease 9 (Cas9) gene-editing technique (Doudna and Charpentier, 2014; Hsu et al., 2014). Specifically targeted by synthetic guide RNA (sgRNA) sequences, Cas9-induced double-stranded breaks typically result in frameshifting insertion/deletions (indels) owing to the action of non-homologous end-joining (NHEJ) DNA repair mechanisms, leading to disruption of the encoded protein.

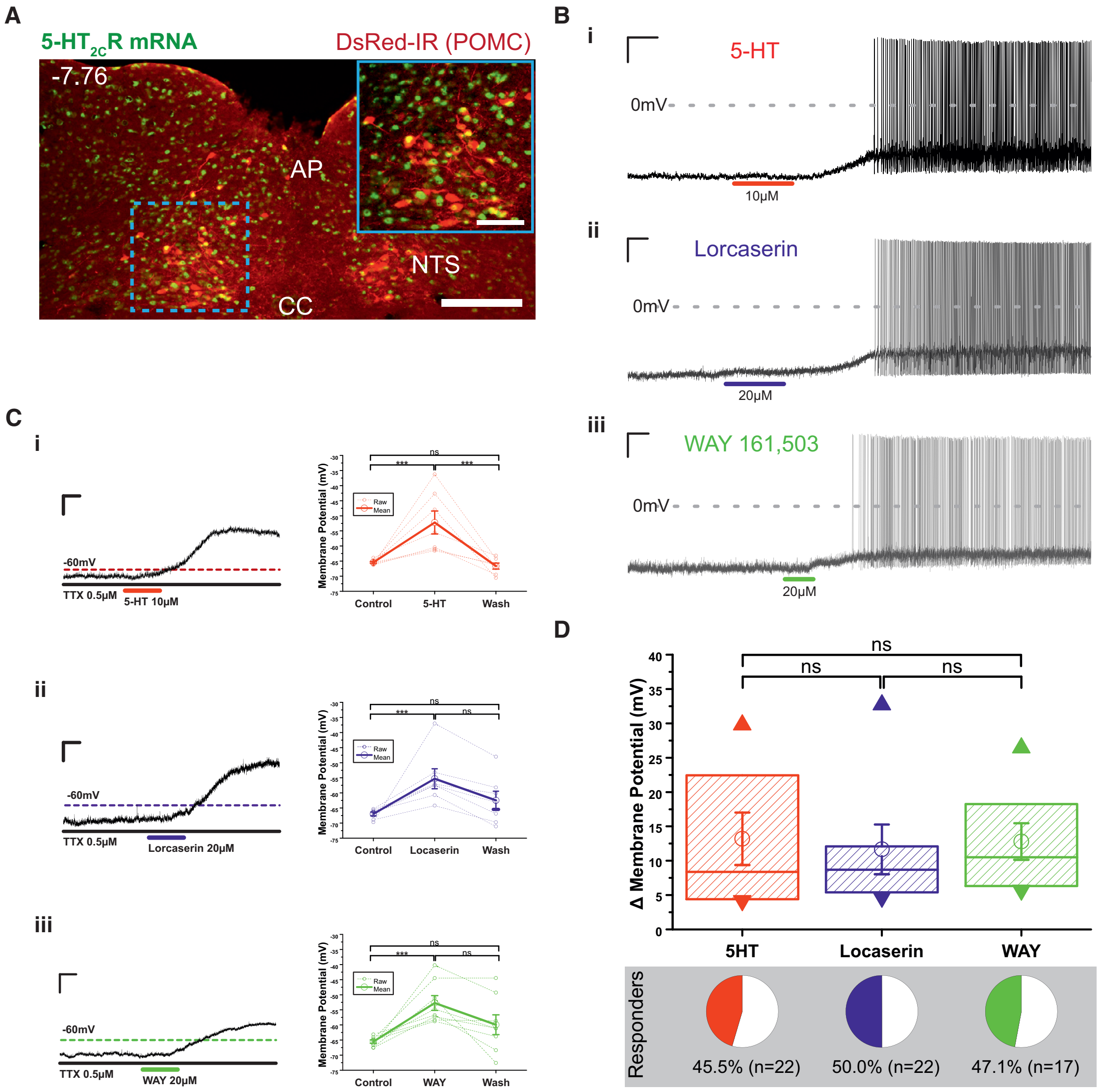

Figure 3. 5-HT and 5-HT$_{2C}$R Agonists Directly Activate POMC^{NTS} Neurons

(A) Representative image illustrating 38% of DsRED-positive POMC^{NTS} neurons (red) express *5-HT$_{2C}$R* mRNA (green; co-expressed, yellow) in *Pomc*^{DsRED} mice. Scale bar, 200 μm; inset scale bar, 50 μm.

(B) Representative current-clamp recordings of POMC^{NTS} neurons in slices from *Pomc*^{DsRED} mice following (i) 10 μM 5-HT, (ii) 20 μM lorcaserin, (iii) and 20 μM WAY161,503.

(C) (i) 5-HT (n = 7; $F_{2,18}$ = 12.9, p = 0.001), (ii) lorcaserin (n = 7; $F_{2,18}$ = 8.2, p = 0.006), and (iii) WAY161,503 (n = 8; $F_{2,21}$ = 10.3, p = 0.002) elicit a significant and reversible change in membrane potential (recorded in 500 nM TTX and from an initial membrane potential of −65 mV, a value achieved through the injection of DC current). Thick lines represent mean ± SEM; dashed lines, raw data. ***p < 0.001.

(D) Summary of membrane potential changes induced by 5-HT, lorcaserin, and WAY161,503. Boxes represent 25th, 50th, and 75th percentile with superimposed mean ± SEM and maximum and minimum values.

NTS, nucleus of the solitary tract; AP, area postrema; CC, central canal.

Following an *in vitro* screening of selected sgRNAs targeting the third exon of the *Pomc* gene, Cas9-induced indels at the predicted positions were detected using a T7 endonuclease I assay (Figure 6A). Two active sgRNAs were cloned into an AAV vector that also expressed eGFP to enable site-specific brain delivery (Swiech et al., 2015).

AAVs expressing *Pomc*-targeting Cas9 and sgRNA-eGFP (*Pomc*$^{ARC\text{-}CRISPR}$) or AAVs expressing sgRNA-eGFP alone (*Pomc*$^{ARC\text{-}WT}$) were then stereotactically injected into the ARC (Figure 6B). Quantitative analysis of hypothalamic α-MSH content revealed *Pomc*$^{ARC\text{-}CRISPR}$ mice had an approximately 60% reduction in α-MSH (Figure 6C). To control for the occurrence of CRISPR/Cas9-induced indels, we implemented a method allowing *ex vivo* detection of DNA heteroduplexes. This is a widely used proxy of indel occurrence *in vitro*. Genomic DNA from *Pomc*$^{ARC\text{-}CRISPR}$ and *Pomc*$^{ARC\text{-}WT}$ mice was extracted and the

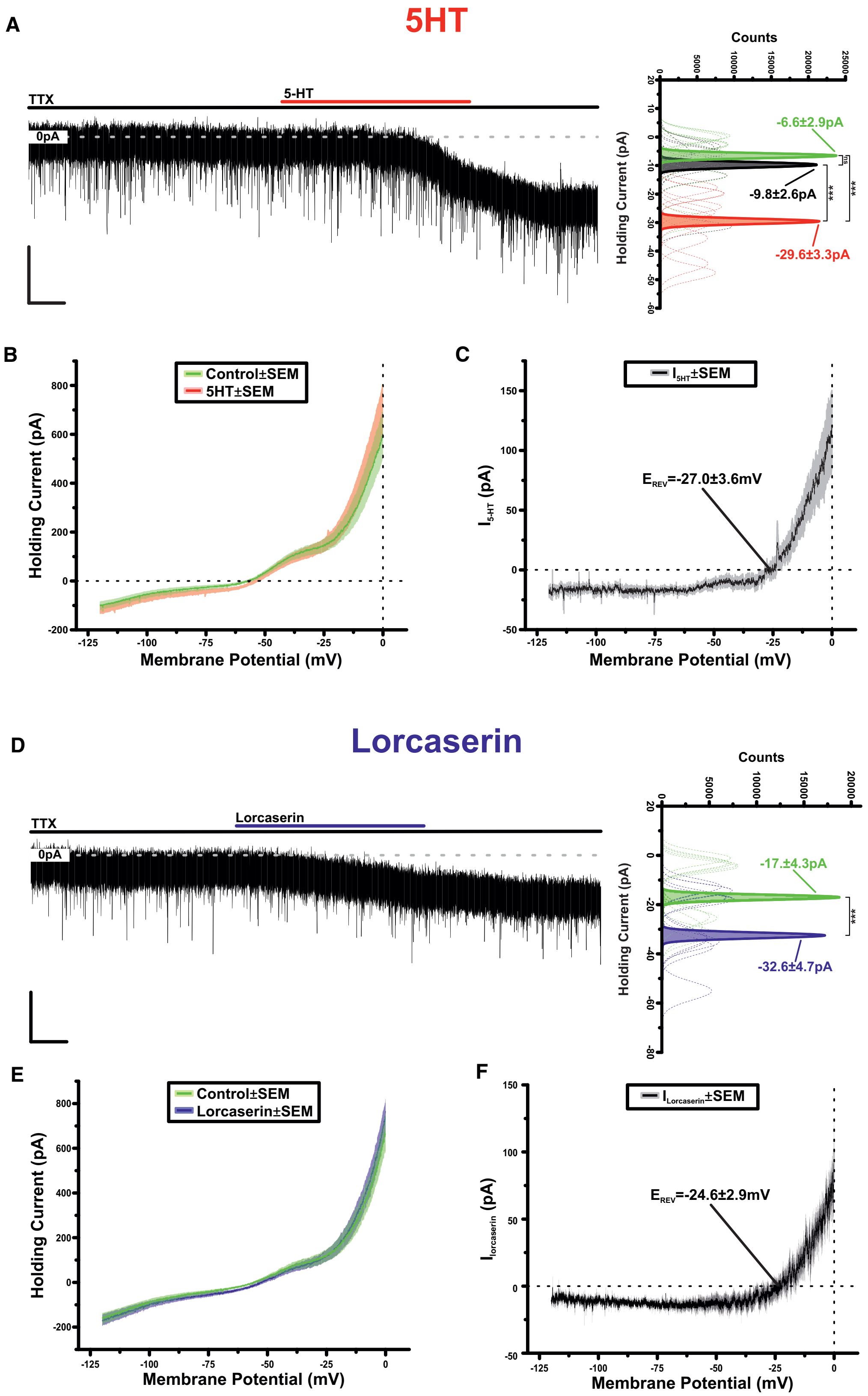

(legend on next page)

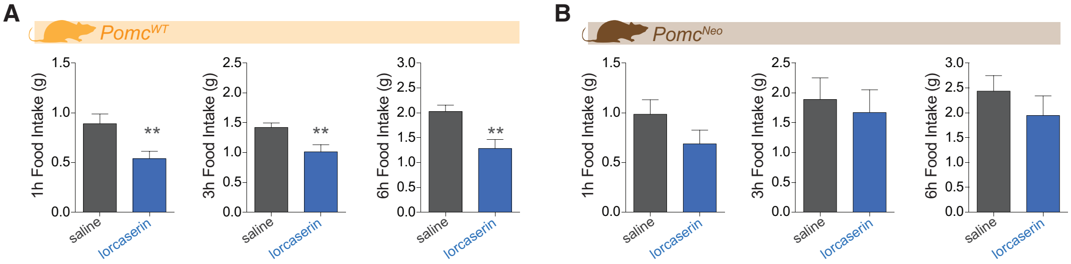

Figure 5. Brain POMC Is a Necessary Neurochemical Mediating 5-HT$_{2C}$R Agonist Feeding Effects

(A) Male and female wild-type mice (*Pomc*^{WT}) treated with lorcaserin (7 mg/kg, i.p.) significantly reduced 1-, 3-, and 6-hr dark cycle food intake compared with saline-treated *Pomc*^{WT} littermates (n = 11 per group; 1 hr: t_{20} = 2.884, p = 0.0092; 3 hr: t_{20} = 2.916, p = 0.0085; 6 hr: t_{20} = 3.373, p = 0.0030).

(B) In contrast, male and female mice with full POMC^{ARC} knockout and partial POMC^{NTS} knockdown (*Pomc*^{Neo}) exhibited a blunted response to lorcaserin treatment as compared with *Pomc*^{Neo} littermates treated with saline (n = 6–7 per group; 1 hr: t_{11} = 1.3204, p = 0.216; 3 hr: t_{11} = 0.4098, p = 0.6898; 6 hr: t_{11} = 0.9486, p = 0.3632).

Data are presented as mean ± SEM. **p < 0.01.

third exon of the *Pomc* gene was amplified by PCR. Subsequent PAGE analysis revealed that DNA heteroduplexes were exclusively in samples obtained from the mediobasal hypothalamus in *Pomc*$^{ARC\text{-}CRISPR}$ mice. DNA heteroduplexes were not found in the hypothalamus of *Pomc*$^{ARC\text{-}WT}$ mice (Figure 6D). Behaviorally, *Pomc*$^{ARC\text{-}CRISPR}$ mice displayed phenotypes consistent with reduced POMC^{ARC} function, including hyperphagia following food deprivation (Figure 6E) and greater operant responding for palatable food (Figure 6F). Next, we assessed the role of POMC^{ARC} in lorcaserin's effects on food intake. As expected, lorcaserin significantly reduced *ad libitum* dark cycle food intake compared with saline treatment in *Pomc*$^{ARC\text{-}WT}$ mice when analyzed over the first 3 hr of the dark cycle (Figure 6G, left) and as cumulative 3 and 6 hr of food intake (Figure 6G, right). In contrast, *Pomc*$^{ARC\text{-}CRISPR}$ mice only significantly responded to the acute anorectic effect of lorcaserin (Figure 6H, left). Analysis of 3- and 6-hr cumulative intake revealed that *Pomc*$^{ARC\text{-}CRISPR}$ mice exhibited an attenuated response to lorcaserin. Specifically, lorcaserin did not significantly alter food intake compared with saline treatment at either time point in *Pomc*$^{ARC\text{-}CRISPR}$ mice (Figure 6H, right). One interpretation of these data is that POMC^{ARC} is not a principal mediator of lorcaserin's acute effects on food intake; rather, it is necessary for the longer-term effect. However, it is possible that the acute response to lorcaserin in *Pomc*$^{ARC\text{-}CRISPR}$ mice is due to incomplete knockdown of POMC^{ARC} and that the subset remaining is sufficient for lorcaserin to reduce feeding at this earlier time point. Nevertheless, these data indicate that lorcaserin requires functional *Pomc* within the ARC to promote its full effect on food intake.

POMC^{NTS} Is Required for the Acute Effect of 5-HT$_{2C}$R Agonists on Food Intake

We next examined the functional significance of POMC^{NTS} in the therapeutic effects of 5-HT$_{2C}$R agonist obesity medications. We created two cohorts of mice by stereotactically delivering AAVs expressing *Pomc*-targeting Cas9 and sgRNA-eGFP (*Pomc*$^{NTS\text{-}CRISPR}$) or AAVs expressing sgRNA-eGFP alone (*Pomc*$^{NTS\text{-}WT}$) to the NTS (Figures 7A and 7B). Since α-MSH expression from NTS explants is not at a high enough level for reliable measurement, we adopted the method used above for *Pomc*$^{ARC\text{-}CRISPR}$ validation, allowing *ex vivo* detection of DNA heteroduplexes to control for the occurrence of CRISPR/Cas9-induced indels. Genomic DNA from *Pomc*$^{NTS\text{-}CRISPR}$ and *Pomc*$^{NTS\text{-}WT}$ mice was extracted and the third exon of the *Pomc* gene was amplified by PCR. Subsequent PAGE analysis revealed that DNA heteroduplexes were exclusively in samples obtained from the NTS/DMV and not an internal control region in *Pomc*$^{NTS\text{-}CRISPR}$ mice (Figures 7C and 7D). As expected, DNA heteroduplexes were not found in either the NTS/DMV or an internal control region of *Pomc*$^{NTS\text{-}WT}$ mice (Figure 7C). These data suggest that *Pomc* has been successfully targeted in *Pomc*$^{NTS\text{-}CRISPR}$ mice, although this method does not provide quantification of the degree of knockdown. As expected, lorcaserin and WAY161,503 significantly reduced *ad libitum* dark cycle food intake in

Figure 4. 5-HT and 5-HT$_{2C}$R Agonists Activate POMC^{NTS} Neurons Via Post-synaptic, Mixed Cationic Current

(A) Representative voltage-clamp recording of a POMC^{NTS} neuron from *Pomc*^{DsRED} NTS slices in the presence of TTX (500 nM). Application of 5-HT (10 μM) results in an inward current (scale bar, 20 s). To the right, sharing its y axis with the raw trace, are Gaussian fits of averaged (solid lines) holding current frequency distributions in control (green), 5-HT (red), and wash (black). Raw data used to produce averages shown as dashed lines (n = 10, $F_{2,27}$ = 18.1, p = 1.02 × 10^{-5}; post hoc Tukey test, ***p < 0.001).

(B) Averaged voltage-clamp ramps (n = 10) acquired in control (green) and at the peak of response (red). Line denotes mean, shading the SEM.

(C) 5-HT-induced current ($I_{5\text{-}HT}$) obtained by the digital subtraction of the traces displayed in (B). Note the reversal at −27.0 ± 3.6 mV (n = 10). Line denotes mean, shading the SEM.

(D) Representative voltage-clamp recording of a POMC^{NTS} neuron in the presence of TTX. Application of lorcaserin (20 μM) results in an inward current (scale bar, 20 s). To the right, sharing its y axis with the raw trace, are Gaussian fits of averaged (solid lines) holding current frequency distributions in control (green) and lorcaserin (blue). Raw data used to produce averages shown as dashed lines (n = 13, t_{12} = 6.2, p = 4.5 × 10^{-5}, t-test paired, ***p < 0.001).

(E) Averaged voltage-clamp ramps (n = 12) acquired in control (green) and at the peak of response (blue). Line denotes mean, shading the SEM.

(F) Lorcaserin-induced current ($I_{Lorcaserin}$) obtained by the digital subtraction of the traces displayed in (E). Note the reversal at −24.6 ± 2.9 mV (n = 12). Line denotes mean, shading the SEM.

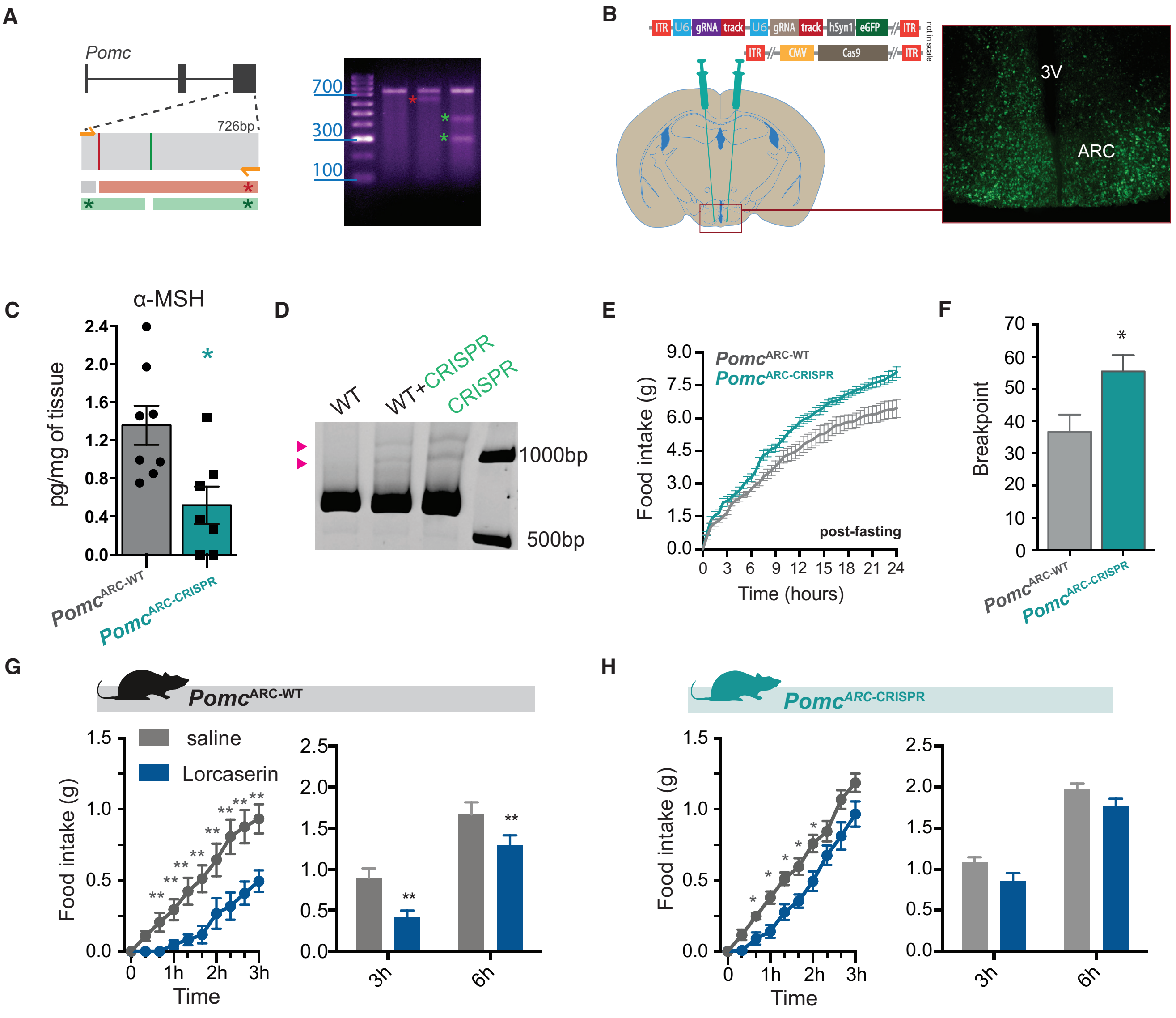

Figure 6. Pomc^{ARC} Is Required for 5-HT$_{2C}$R Agonist Hypophagia

(A) Representative screening of sgRNAs targeting the exon 3 of the *Pomc* gene in Neuro2A cells and indels detection using a T7 endonuclease I assay. (A, left) Sites of potential indels in exon 3 are indicated by red or green bars, and the presence of indels results in the fragments indicated by red or green asterisks, respectively, following endonuclease treatment. (A, right) Bands corresponding to these fragments are indicated with red and green asterisks on the gel.

(B) Schematic of bilateral mediobasal hypothalamic stereotactic delivery of AAVs expressing *Pomc*-targeting CRISPR/Cas9 and EGFP (*Pomc*$^{ARC\text{-}CRISPR}$). Representative image of GFP within the arcuate nucleus of the hypothalamus (ARC) third ventricle (3V).

(C) Hypothalamic content of *Pomc* peptide product α-melanocyte-stimulating hormone (α-MSH) 6 weeks after AAV injection is significantly reduced as measured by quantitative fluorescent EIA assay in *Pomc*$^{ARC\text{-}CRISPR}$ mice compared with controls injected with AAV-expressing sgRNA-eGFP alone (*Pomc*$^{ARC\text{-}WT}$), illustrating that *in vivo* CRISPR targeting knocked down *Pomc* in the ARC ($p = 0.0059$; Mann-Whitney test).

(D) PAGE analysis identified heteroduplexes (pink arrowheads) from exon 3 of *Pomc* amplified from the mediobasal hypothalamus of *Pomc*$^{ARC\text{-}CRISPR}$ mice.

(E) *Pomc*$^{ARC\text{-}CRISPR}$ mice exhibit 24 hr of hyperphagia for home cage chow following food restriction compared with *Pomc*$^{ARC\text{-}WT}$ mice ($n = 7$–8; $F_{1,13} = 11.27$, $p = 0.005$).

(F) Likewise, *Pomc*$^{ARC\text{-}CRISPR}$ mice show greater motivation to work for palatable food compared with *Pomc*$^{ARC\text{-}WT}$ mice as assessed by the breakpoint of lever presses for chocolate pellets using a progressive-ratio schedule ($n = 7$–8; $t_{12} = 2.463$, $p = 0.029$).

(G) (Left) Lorcaserin (7 mg/kg, i.p.) reduced acute food intake within the first 3 hr (tick marks on x axis represent 20-min intervals) of the dark cycle in *Pomc*$^{ARC\text{-}WT}$ mice ($n = 7$; time: $F_{9,54} = 38.05$, $p < 0.0001$; treatment: $F_{1,6} = 39.94$, $p = 0.0007$; interaction: $F_{9,54} = 13.91$, $p < 0.0001$) and (right) as analyzed as total 3- and 6-hr cumulative intake (time: $F_{1,6} = 169.7$, $p < 0.0001$; treatment: $F_{1,6} = 11.22$, $p = 0.0154$; interaction: $F_{1,6} = 0.8351$, $p = 0.3960$).

(H) (Left) Lorcaserin (7 mg/kg, i.p.) also reduced acute food intake within the first 3 hr in *Pomc*$^{ARC\text{-}CRISPR}$ mice ($n = 10$; time: $F_{9,81} = 168.1$, $p < 0.0001$; treatment: $F_{1,9} = 16.46$, $p = 0.0029$; interaction: $F_{9,81} = 2.108$, $p = 0.0381$). (Right) However, lorcaserin was not effective in reducing cumulative food intake compared with saline treatment in *Pomc*$^{ARC\text{-}CRISPR}$ mice when analyzed at 3 or 6 hr food intake with Sidak's post hoc comparisons ($n = 10$; time: $F_{1,9} = 130.8$, $p < 0.0001$; treatment: $F_{1,9} = 110.44$, $p = 0.0103$; interaction: $F_{1,9} = 0.01045$, $p = 0.9208$).

Data are presented as mean ± SEM. Sidak's post hoc comparisons $^{*}p < 0.05$, $^{**}p < 0.01$.

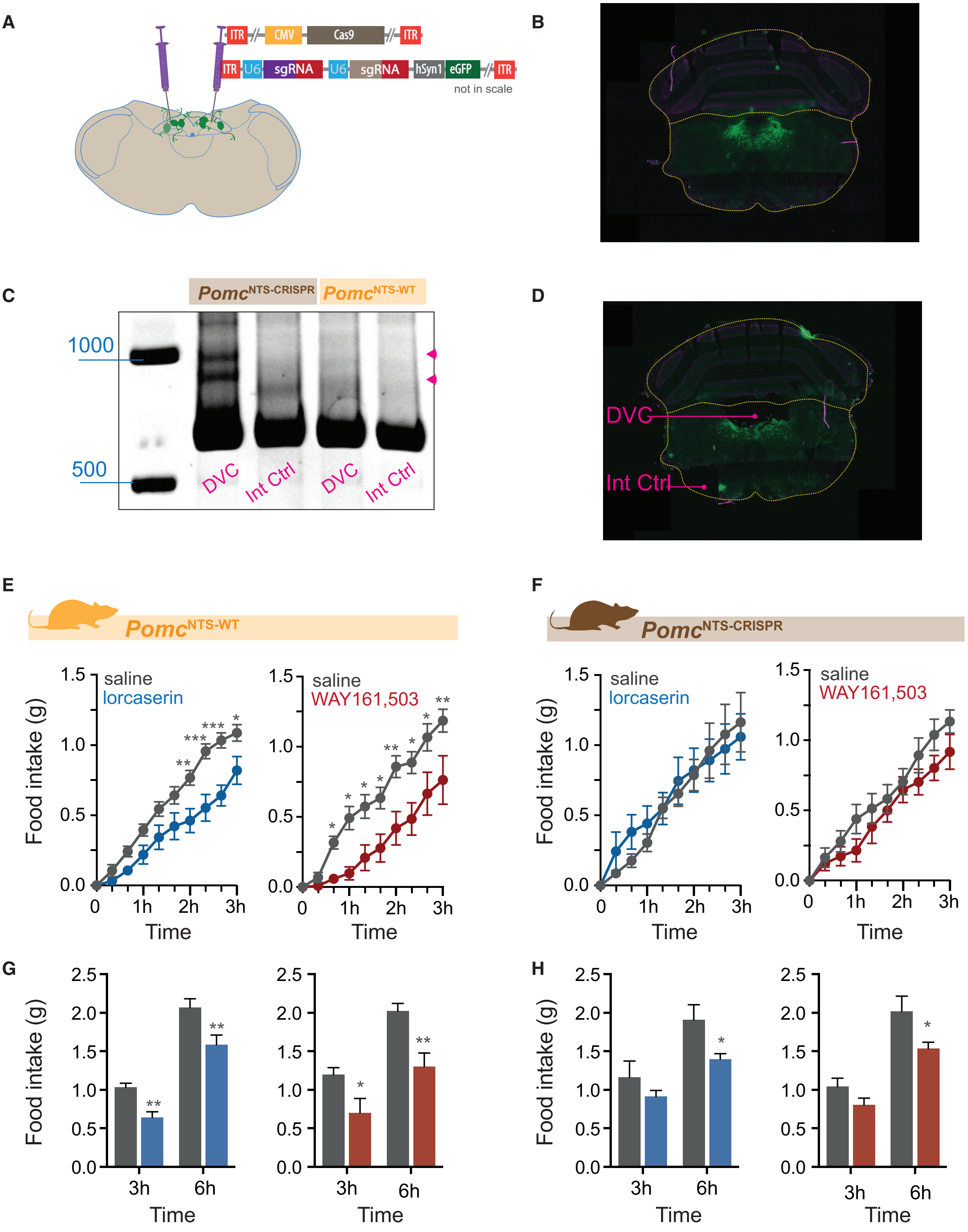

(legend on next page)

Pomc$^{NTS\text{-}WT}$ mice over 6 hr (Figures 7E and 7G). However, following administration of 5-HT_{2C}R agonists, *Pomc*$^{NTS\text{-}CRISPR}$ mice exhibited an attenuated anorectic response to both lorcaserin and WAY161,503 during the first 3 hr of the dark cycle (Figure 7F). This attenuated effect to lorcaserin and WAY161,503 did not persist for the full 6 hr in *Pomc*$^{NTS\text{-}CRISPR}$ mice. Lorcaserin significantly reduced 6-hr cumulative intake in *Pomc*$^{NTS\text{-}CRISPR}$ mice compared with saline treatment (Figure 7H). Taken together with the *Pomc*^{NEO} and *Pomc*$^{ARC\text{-}CRISPR}$ results, these data suggest that POMC^{NTS} is required for the acute effect of lorcaserin on food intake, whereas POMC^{ARC} is required for the longer-term effect on feeding. These findings are generally consistent with previous reports using chemogenetic and optogenetic approaches to examine POMC neuron function, which suggest that POMC^{ARC} and POMC^{NTS} neurons affect feeding on different time scales (Aponte et al., 2011; Koch et al., 2015; Zhan et al., 2013).

DISCUSSION

Obesity negatively affects human health on a global scale. If we are to meet this challenge and design new medications, it is paramount that we gain a more complete understanding of the mechanisms regulating food intake and body weight. The 5-HT_{2C}R agonist lorcaserin is a current obesity medication; however, neither the specific subset of 5-HT_{2C}Rs coordinating the therapeutic effect nor the neurochemical mediator targeted by these receptors has been fully defined. Earlier research genetically manipulating the receptor revealed that the subset of 5-HT_{2C}Rs co-expressed with brain POMC are both sufficient (Xu et al., 2010) and necessary (Berglund et al., 2013) to modulate the anorectic effect of preclinical 5-HT_{2C}R agonists. Given that 5-HT_{2C}R agonists increase the activity of POMC^{ARC} neurons (Burke and Heisler, 2015; Doslikova et al., 2013; Heisler et al., 2002), it was presumed that these neurons were the mediator of this effect. However, in light of recent findings that POMC^{ARC} neurons suppress appetite on a time scale of hours (Aponte et al., 2011; Koch et al., 2015; Zhan et al., 2013), we considered whether 5-HT_{2C}Rs outside the ARC are necessary for 5-HT_{2C}R's acute effects on feeding. Here we identify the function of a previously uncharacterized subset of 5-HT_{2C}R expressing neurons, localized within the NTS, and reveal that the activation of this subpopulation has a significant and rapid impact on feeding behavior, and furthermore is sufficient to drive lorcaserin's acute reduction in food intake. In addition, we report that a subset of POMC^{NTS} neurons express 5-HT_{2C}Rs and that 5-HT and 5-HT_{2C}R agonists directly activate POMC^{NTS} neurons via a post-synaptic, mixed cationic current. We also clarify that brain POMC is necessary for lorcaserin to influence feeding. Although POMC is not abundantly expressed within the NTS, we report that POMC in this region is required for 5-HT_{2C}R agonist obesity medications to acutely reduce food intake. These data thereby reveal a subpopulation of 5-HT_{2C}Rs, which when pharmacologically activated significantly reduce food intake, identify that the mechanistic underpinnings of this acute effect is via POMC^{NTS}, and demonstrate that this discrete 5-HT_{2C}R^{NTS} subpopulation is therapeutically relevant to obesity medications targeting the 5-HT_{2C}Rs.

Limitations of Study

It is possible that the results obtained are affected by incomplete knockdown of *Pomc* in the ARC and NTS. Although altering POMC^{NTS} expression significantly attenuated the acute effect of lorcaserin on food intake, at baseline *Pomc*$^{NTS\text{-}CRISPR}$ mice displayed food intake comparable with *Pomc*$^{NTS\text{-}WT}$ mice during *ad libitum* feeding (Figures 7E and 7F; and data not shown). These data are consistent with a previous report of diphtheria toxin POMC^{NTS} cell ablation in a *Pomc*^{Cre} mouse line, which did not produce an effect on *ad libitum* food intake (Zhan et al., 2013). However, we cannot exclude the possibility that this absence of baseline phenotype *Pomc*$^{NTS\text{-}CRISPR}$ mice is due to incomplete knockdown of *Pomc*^{NTS}. Further studies are required to fully interrogate the physiological role of POMC^{NTS}. Nevertheless, our data reveal that the relatively small source of POMC specifically derived within the NTS is necessary for the obesity medication lorcaserin and the preclinical compound WAY161,503 to promote their full effects on feeding behavior.

STAR★METHODS

Detailed methods are provided in the online version of this paper and include the following:

- KEY RESOURCES TABLE
- CONTACT FOR REAGENT AND RESOURCE SHARING

Figure 7. Pomc^{NTS} Is Required for 5-HT_{2C}R Agonist Hypophagia

(A) AAV-mediated strategy to express in the nucleus of the solitary tract (NTS) *Pomc*-targeting sgRNAs and Cas9. Schematic of bilateral NTS stereotactic delivery of AAVs expressing *Pomc*-targeting CRISPR/Cas9 and EGFP (*Pomc*$^{NTS\text{-}CRISPR}$).

(B) Representative image of eGFP expression within the dorsal vagal complex (DVC).

(C) PAGE analysis identified heteroduplexes (pink arrowheads) from exon 3 of *Pomc* amplified from the DVC of *Pomc*$^{NTS\text{-}CRISPR}$ mice.

(D) Representative image of tissue samples taken from the NTS and broader dorsal vagal complex (DVC) and an internal control region (Int Ctrl).

(E and F) 5-HT_{2C}R agonists lorcaserin and WAY161,503 (7 mg/kg, i.p.) reduced acute food intake in control *Pomc*$^{NTS\text{-}WT}$ mice (E) within the first 3 hr (tick marks on x axis represent 20-min intervals) of the dark cycle (lorcaserin, n = 8; time: $F_{9,63} = 102.2$, $p < 0.0001$; treatment: $F_{1,7} = 18.55$, $p = 0.0035$; interaction: $F_{9,63} = 6.245$, $p < 0.001$. WAY161,503, n = 8; time: $F_{9,63} = 54.95$, $p < 0.0001$; treatment: $F_{1,7} = 24.28$, $p = 0.0017$; interaction: $F_{9,63} = 3.369$, $p = 0.0020$), but not in *Pomc*$^{NTS\text{-}CRISPR}$ mice (F) (lorcaserin, n = 7; time: $F_{9,54} = 38.39$, $p < 0.0001$; treatment: $F_{1,6} = 0.1580$, $p = 0.7048$; interaction: $F_{9,54} = 0.2533$, $p = 0.9841$. WAY161,503, n = 7; time: $F_{9,54} = 62.69$, $p < 0.0001$; treatment: $F_{1,6} = 2.444$, $p = 0.1690$; interaction: $F_{9,54} = 0.7558$, $p = 0.6567$).

(G) Likewise, when analyzed as total cumulative 3- and 6-hr dark cycle intake, lorcaserin and WAY161,503 significantly reduced food intake in control *Pomc*$^{NTS\text{-}WT}$ mice (lorcaserin, n = 8; time: $F_{1,7} = 96.71$, $p < 0.0001$; treatment: $F_{1,7} = 70.40$, $p < 0.0001$; interaction: $F_{1,7} = 0.6355$, $p = 0.4515$. WAY161,503, n = 8; time: $F_{1,7} = 62.88$, $p < 0.0001$; treatment: $F_{1,7} = 17.53$, $p = 0.0041$; interaction: $F_{1,7} = 4.238$, $p = 0.0785$).

(H) POMC^{NTS} knockdown in *Pomc*$^{NTS\text{-}CRISPR}$ mice prevented the acute 3-hr anorectic effect of 5-HT_{2C}R agonists, but was not sufficient to prevent the full effect 6 hr post treatment as analyzed with Sidak's post hoc comparisons (lorcaserin, n = 7; time: $F_{1,6} = 92.76$, $p < 0.0001$; treatment: $F_{1,6} = 2.823$, $p = 0.1439$; interaction: $F_{1,6} = 5.309$, $p = 0.0608$. WAY161,503, n = 7; time: $F_{1,6} = 66.68$, $p = 0.0002$; treatment: $F_{1,6} = 4.152$, $p = 0.00877$; interaction: $F_{1,6} = 4.005$, $p = 0.0923$). Data are presented as mean ± SEM. Sidak's post hoc comparisons *p < 0.05, **p < 0.01, ***p < 0.001.

- EXPERIMENTAL MODEL AND SUBJECT DETAILS
  - Mice
- METHOD DETAILS
  - Stereotaxic Surgery and Viral Vectors
  - Feeding Studies
  - Immunohistochemistry (IHC) and Fluorescent In Situ Hybridization Histochemistry (FISH)
  - Electrophysiology and Chemogenetic Validation Analysis
  - Drugs
- QUANTIFICATION AND STATISTICAL ANALYSIS

SUPPLEMENTAL INFORMATION

Supplemental Information includes two figures and can be found with this article online at https://doi.org/10.1016/j.cmet.2018.07.017.

ACKNOWLEDGMENTS

The authors wish to thank members of staff of the Medical Research Facility, University of Aberdeen, Ms. Raffaella Chianese and Dr. Susan Jalicy, for technical assistance. PX330 and PX552 plasmids were a gift from Prof. Feng Zhang (Massachusetts Institute of Technology, Massachusetts, USA). DREADD vectors were a gift from Prof. Bryan Roth (University of North Carolina at Chapel Hill, North Carolina, USA). $Pomc^{DsRED}$ and $Pomc^{NEO}$ mice were a gift from Prof. Malcolm Low (University of Michigan, Michigan, USA). Codes to analyze operant-responding for food were a gift from Dr. Vladimir Orduña Trujillo (National Autonomous University of Mexico, Mexico). This work was supported by the Wellcome Trust (L.K.H.; WT098012), Wellcome Trust and the University of Aberdeen (G.D.; 105625/Z/14/Z), Biotechnology and Biological Sciences Research Council (L.K.H., BB/K001418/1, BB/N017838/1; and J.J.R., BB/K017772/1), Medical Research Council (J.J.R., MR/L002620/1; G.D., MR/P009824/1; L.K.H., J.J.R., G.D., MC/PC/15077), British Society of Neuroendocrinology (G.D.), NIH and the Marilyn H. Vincent Foundation (M.G.M.; DK056731, DK034933).

AUTHOR CONTRIBUTIONS

G.D. and L.K.H. conceived the study and designed experiments. G.D. performed experiments with help from B.D., C.O.-S., P.B.M.d.M., T.G., and C. Cristiano. M.L. and J.J.R. performed cell-culture experiments. C. Cansell performed fluorescence *in situ* hybridization experiments. L.K.B. and B.D. performed studies with $Pomc^{NEO}$ mice. M.G.-Y. and M.G.M. generated $5\text{-}HT_{2C}R^{Cre}$ mice. D.L. performed electrophysiology experiments. G.D., D.L., and L.K.H. wrote the manuscript with input from all other authors.

DECLARATIONS OF INTERESTS

L.K.H. has consulted for Eisai, Inc.

Received: November 15, 2016
Revised: February 12, 2018
Accepted: July 27, 2018
Published: August 23, 2018

REFERENCES

Aponte, Y., Atasoy, D., and Sternson, S.M. (2011). AGRP neurons are sufficient to orchestrate feeding behavior rapidly and without training. Nat. Neurosci. *14*, 351–355.

Appleyard, S.M., Bailey, T.W., Doyle, M.W., Jin, Y.H., Smart, J.L., Low, M.J., and Andresen, M.C. (2005). Proopiomelanocortin neurons in nucleus tractus solitarius are activated by visceral afferents: regulation by cholecystokinin and opioids. J. Neurosci. *25*, 3578–3585.

Armbruster, B.N., Li, X., Pausch, M.H., Herlitze, S., and Roth, B.L. (2007). Evolving the lock to fit the key to create a family of G protein-coupled receptors potently activated by an inert ligand. Proc. Natl. Acad. Sci. USA *104*, 5163–5168.

Aronne, L., Shanahan, W., Fain, R., Glicklich, A., Soliman, W., Li, Y., and Smith, S. (2014). Safety and efficacy of lorcaserin: a combined analysis of the BLOOM and BLOSSOM trials. Postgrad. Med. *126*, 7–18.

Berglund, E.D., Liu, C., Sohn, J.W., Liu, T., Kim, M.H., Lee, C.E., Vianna, C.R., Williams, K.W., Xu, Y., and Elmquist, J.K. (2013). Serotonin 2C receptors in pro-opiomelanocortin neurons regulate energy and glucose homeostasis. J. Clin. Invest. *123*, 5061–5070.

Breisch, S.T., Zemlan, F.P., and Hoebel, B.G. (1976). Hyperphagia and obesity following serotonin depletion by intraventricular p-chlorophenylalanine. Science *192*, 382–385.

Bumaschny, V.F., Yamashita, M., Casas-Cordero, R., Otero-Corchon, V., de Souza, F.S., Rubinstein, M., and Low, M.J. (2012). Obesity-programmed mice are rescued by early genetic intervention. J. Clin. Invest. *122*, 4203–4212.

Burke, L.K., Doslikova, B., D'Agostino, G., Garfield, A.S., Farooq, G., Burdakov, D., Low, M.J., Rubinstein, M., Evans, M.L., Billups, B., et al. (2014). 5-HT obesity medication efficacy via POMC activation is maintained during aging. Endocrinology *155*, 3732–3738.

Burke, L.K., Doslikova, B., D'Agostino, G., Greenwald-Yarnell, M., Georgescu, T., Chianese, R., Martinez de Morentin, P.B., Ogunnowo-Bada, E., Cansell, C., Valencia-Torres, L., et al. (2016). Sex difference in physical activity, energy expenditure and obesity driven by a subpopulation of hypothalamic POMC neurons. Mol. Metab. *5*, 245–252.

Burke, L.K., and Heisler, L.K. (2015). 5-hydroxytryptamine medications for the treatment of obesity. J. Neuroendocrinol. *27*, 389–398.

Burke, L.K., Ogunnowo-Bada, E., Georgescu, T., Cristiano, C., de Morentin, P.B.M., Valencia Torres, L., D'Agostino, G., Riches, C., Heeley, N., Ruan, Y., et al. (2017). Lorcaserin improves glycemic control via a melanocortin neurocircuit. Mol. Metab. *6*, 1092–1102.

D'Agostino, G., and Diano, S. (2010). Alpha-melanocyte stimulating hormone: production and degradation. J. Mol. Med. (Berl.) *88*, 1195–1201.

D'Agostino, G., Lyons, D.J., Cristiano, C., Burke, L.K., Madara, J.C., Campbell, J.N., Garcia, A.P., Land, B.B., Lowell, B.B., Dileone, R.J., et al. (2016). Appetite controlled by a cholecystokinin nucleus of the solitary tract to hypothalamus neurocircuit. eLife *5*, https://doi.org/10.7554/eLife.12225.

Doslikova, B., Garfield, A.S., Shaw, J., Evans, M.L., Burdakov, D., Billups, B., and Heisler, L.K. (2013). 5-HT2C receptor agonist anorectic efficacy potentiated by 5-HT1B receptor agonist coapplication: an effect mediated via increased proportion of pro-opiomelanocortin neurons activated. J. Neurosci. *33*, 9800–9804.

Doudna, J.A., and Charpentier, E. (2014). Genome editing. The new frontier of genome engineering with CRISPR-Cas9. Science *346*, 1258096.

Fan, W., Ellacott, K.L., Halatchev, I.G., Takahashi, K., Yu, P., and Cone, R.D. (2004). Cholecystokinin-mediated suppression of feeding involves the brainstem melanocortin system. Nat. Neurosci. *7*, 335–336.

Fletcher, P.J., Tampakeras, M., Sinyard, J., Slassi, A., Isaac, M., and Higgins, G.A. (2009). Characterizing the effects of 5-HT(2C) receptor ligands on motor activity and feeding behaviour in 5-HT(2C) receptor knockout mice. Neuropharmacology *57*, 259–267.

Grill, H.J., and Hayes, M.R. (2009). The nucleus tractus solitarius: a portal for visceral afferent signal processing, energy status assessment and integration of their combined effects on food intake. Int. J. Obes. (Lond.) *33* (*Suppl 1*), S11–S15.

Heisler, L.K., Cowley, M.A., Tecott, L.H., Fan, W., Low, M.J., Smart, J.L., Rubinstein, M., Tatro, J.B., Marcus, J.N., Holstege, H., et al. (2002). Activation of central melanocortin pathways by fenfluramine. Science *297*, 609–611.

Heisler, L.K., Jobst, E.E., Sutton, G.M., Zhou, L., Borok, E., Thornton-Jones, Z., Liu, H.Y., Zigman, J.M., Balthasar, N., Kishi, T., et al. (2006). Serotonin reciprocally regulates melanocortin neurons to modulate food intake. Neuron *51*, 239–249.

Hentges, S.T., Otero-Corchon, V., Pennock, R.L., King, C.M., and Low, M.J. (2009). Proopiomelanocortin expression in both GABA and glutamate neurons. J. Neurosci. *29*, 13684–13690.

Hsu, P.D., Lander, E.S., and Zhang, F. (2014). Development and applications of CRISPR-Cas9 for genome engineering. Cell *157*, 1262–1278.

Joseph, S.A., Pilcher, W.H., and Bennett-Clarke, C. (1983). Immunocytochemical localization of ACTH perikarya in nucleus tractus solitarius: evidence for a second opiocortin neuronal system. Neurosci. Lett. *38*, 221–225.

Julius, D., MacDermott, A.B., Axel, R., and Jessell, T.M. (1988). Molecular characterization of a functional cDNA encoding the serotonin 1c receptor. Science *241*, 558–564.

Koch, M., Varela, L., Kim, J.G., Kim, J.D., Hernandez-Nuno, F., Simonds, S.E., Castorena, C.M., Vianna, C.R., Elmquist, J.K., Morozov, Y.M., et al. (2015). Hypothalamic POMC neurons promote cannabinoid-induced feeding. Nature *519*, 45–50.

Krashes, M.J., Koda, S., Ye, C., Rogan, S.C., Adams, A.C., Cusher, D.S., Maratos-Flier, E., Roth, B.L., and Lowell, B.B. (2011). Rapid, reversible activation of AgRP neurons drives feeding behavior in mice. J. Clin. Invest. *121*, 1424–1428.

Lam, D.D., Przydzial, M.J., Ridley, S.H., Yeo, G.S., Rochford, J.J., O'Rahilly, S., and Heisler, L.K. (2008). Serotonin 5-HT2C receptor agonist promotes hypophagia via downstream activation of melanocortin 4 receptors. Endocrinology *149*, 1323–1328.

Lam, D.D., Zhou, L., Vegge, A., Xiu, P.Y., Christensen, B.T., Osundiji, M.A., Yueh, C.Y., Evans, M.L., and Heisler, L.K. (2009). Distribution and neurochemical characterization of neurons within the nucleus of the solitary tract responsive to serotonin agonist-induced hypophagia. Behav. Brain Res. *196*, 139–143.

Marcinkiewcz, C.A., Mazzone, C.M., D'Agostino, G., Halladay, L.R., Hardaway, J.A., DiBerto, J.F., Navarro, M., Burnham, N., Cristiano, C., Dorrier, C.E., et al. (2016). Serotonin engages an anxiety and fear-promoting circuit in the extended amygdala. Nature *537*, 97–101.

Nonogaki, K., Abdallah, L., Goulding, E.H., Bonasera, S.J., and Tecott, L.H. (2003). Hyperactivity and reduced energy cost of physical activity in serotonin 5-HT(2C) receptor mutant mice. Diabetes *52*, 315–320.

Olarte-Sánchez, C.M., Valencia Torres, L., Body, S., Cassaday, H.J., Bradshaw, C.M., and Szabadi, E. (2012). Effect of orexin-B saporin-induced lesions of the lateral hypothalamus on performance on a progressive ratio schedule. J. Psychopharmacol. *26*, 871–886.

Richardson, N.R., and Roberts, D.C. (1996). Progressive ratio schedules in drug self-administration studies in rats: a method to evaluate reinforcing efficacy. J. Neurosci. Methods *66*, 1–11.

Smith, S.R., Weissman, N.J., Anderson, C.M., Sanchez, M., Chuang, E., Stubbe, S., Bays, H., and Shanahan, W.R. (2010). Multicenter, placebo-controlled trial of lorcaserin for weight management. N. Engl. J. Med. *363*, 245–256.

Sohn, J.W., Xu, Y., Jones, J.E., Wickman, K., Williams, K.W., and Elmquist, J.K. (2011). Serotonin 2C receptor activates a distinct population of arcuate pro-opiomelanocortin neurons via TRPC channels. Neuron *71*, 488–497.

Stark, J.A., Davies, K.E., Williams, S.R., and Luckman, S.M. (2006). Functional magnetic resonance imaging and c-Fos mapping in rats following an anorectic dose of m-chlorophenylpiperazine. Neuroimage *31*, 1228–1237.

Swiech, L., Heidenreich, M., Banerjee, A., Habib, N., Li, Y., Trombetta, J., Sur, M., and Zhang, F. (2015). In vivo interrogation of gene function in the mammalian brain using CRISPR-Cas9. Nat. Biotechnol. *33*, 102–106.

Tecott, L.H., Sun, L.M., Akana, S.F., Strack, A.M., Lowenstein, D.H., Dallman, M.F., and Julius, D. (1995). Eating disorder and epilepsy in mice lacking 5-HT2c serotonin receptors. Nature *374*, 542–546.

Wade, J.M., Juneja, P., MacKay, A.W., Graham, J., Havel, P.J., Tecott, L.H., and Goulding, E.H. (2008). Synergistic impairment of glucose homeostasis in ob/ob mice lacking functional serotonin 2C receptors. Endocrinology *149*, 955–961.

Williams, K.W., Scott, M.M., and Elmquist, J.K. (2011). Modulation of the central melanocortin system by leptin, insulin, and serotonin: co-ordinated actions in a dispersed neuronal network. Eur. J. Pharmacol. *660*, 2–12.

Xu, Y., Jones, J.E., Kohno, D., Williams, K.W., Lee, C.E., Choi, M.J., Anderson, J.G., Heisler, L.K., Zigman, J.M., Lowell, B.B., et al. (2008). 5-HT2CRs expressed by pro-opiomelanocortin neurons regulate energy homeostasis. Neuron *60*, 582–589.

Xu, Y., Jones, J.E., Lauzon, D.A., Anderson, J.G., Balthasar, N., Heisler, L.K., Zinn, A.R., Lowell, B.B., and Elmquist, J.K. (2010). A serotonin and melanocortin circuit mediates D-fenfluramine anorexia. J. Neurosci. *30*, 14630–14634.

Zhan, C., Zhou, J., Feng, Q., Zhang, J.E., Lin, S., Bao, J., Wu, P., and Luo, M. (2013). Acute and long-term suppression of feeding behavior by POMC neurons in the brainstem and hypothalamus, respectively. J. Neurosci. *33*, 3624–3632.

STAR★METHODS

KEY RESOURCES TABLE

REAGENT or RESOURCE	SOURCE	IDENTIFIER
Antibodies		
Chicken anti-GFP	AbCam	Cat# ab13970; RRID: AB_300798
Rabbit anti-dsRED	Rockland	Cat# 600-401-379; RRID: RRID: AB_2209751
Rabbit anti-c-FOS	Calbiochem	Cat# PC38; RRID:AB_2106755
Goat mCherry (RFP)	Sicgen	Cat# AB0040-200; RRID:AB_2333092
Donkey polyclonal anti-chicken alexa 488	Jackson ImmunoResearch	Cat# 703-545-155; RRID: AB_2340375
Goat anti-Rabbit IgG Secondary Antibody, Alexa Fluor 594	Life Technologies	Cat# A-11012, RRID:AB_141359
Donkey anti-Goat IgG Secondary Antibody, Alexa Fluor 594	Life Technologies	Cat# A-11057; RRID:AB_142581
Biotin-SP-AffiniPure F(ab')2 Fragment Donkey Anti-Rabbit IgG	Jackson ImmunoResearch	Cat# 711-066-152, RRID:AB_2340594
Streptavidin, Alexa Fluor® 568 conjugate antibody	Life Technologies	Cat# S-11226, RRID:AB_2315774
Bacterial and Virus Strains		
One Shot® Stbl3™ Chemically Competent *E. coli*	Invitrogen	Cat# C737303
AAV8-hSyn-DIO-hM3D$_q$-mCherry	University North Carolina Vector Core	N/A
AAV8-hSyn-DIO-hM4D$_i$-mCherry	University North Carolina Vector Core	N/A
AAV8-hSyn-mCherry-Cre	University North Carolina Vector Core	N/A
AAV-U6sgRNA(SapI)_hSyn-GFP-KASH-bGH	Unitat de Producció de Vectors, Universitat Autonoma Barcelona, Spain	N/A
AAV/DJ-CMV-spCas9	Vector Biolabs	Cat# 7120
Chemicals, Peptides, and Recombinant Proteins		
Clozapine-N-oxide (CNO)	Tocris Bioscience	Cat. No. 4936; CAS: 34233-69-7
Lorcaserin Hydrochloride	LGM Pharma	Cat# 846589-98-8 Lot# 20130115
WAY 161503 hydrochloride	Tocris Bioscience	Cat. No. 1801; CAS: 276695-22-8
Critical Commercial Assays		
In-Fusion HD cloning Kit	Clonotech Laboratories	Cat No. 638910
Plasmid plus Maxi kit	QIAGEN	Cat No. 12943
QIAquick Gel Extraction Kit	QIAGEN	Cat No. 28704
FuGENE HD Transfection Reagent	Promega	Cat No E2311
PureLink Genomic DNA Mini Kit	Invitrogen	Cat No K182001
Surveyor Mutation Detection Kit	Integrated DNA Technologies	Cat No. 706020
MSH, alpha EIA kit	Phoenix Pharmaceutical Inc.	Cat No FEK-043-01
Experimental Models: Cell Lines		
Neuro-2a (N2a) cells	ATCC	CCL-131
Experimental Models: Organisms/Strains		
Mouse: *5-HT$_{2C}$R-ires^{Cre}*	Burke et al., 2016; Marcinkiewcz et al., 2016	N/A
Mouse: *POMC^{dsRed}*	Hentges et al., 2009	N/A
Mouse: *POMC^{NEO}*	Bumaschny et al., 2012	N/A
Mouse: *loxtb5-HT$_{2C}$R* (B6.129-Htr2c^{tm1Jke}/J)	Jackson Laboratory	JAX: 015821
Mouse: C57BL/6J	Jackson Laboratory	JAX: 000664

(*Continued on next page*)

REAGENT or RESOURCE	SOURCE	IDENTIFIER
Continued		
Oligonucleotides		
Genomic DNA *Pomc* PCR primer F: GCTTGCATCCGGGCTTGC	This paper	N/A
genomic DNA *Pomc* PCR primer R: GACTTTATTTACGCAGTTTTTATTGAAGATCAGAGC	This paper	N/A
Pomc gRNA1: GGTGGGCAAGAAACGGCGCC	This paper	N/A
Pomc gRNA2: GTGACCCATGACGTACTTCCG	This paper	N/A
Recombinant DNA		
pX330-U6-Chimeric_BB-CBh-hSpCas9 (PX330)	Addgene	Addgene Plasmid #42230
pAAV-U6sgRNA(SapI)_hSyn-GFP-KASH-bGH (PX552)	Addgene	Addgene Plasmid #60958
cDNA HTR2C cDNA	Julius et al., 1988	N/A
Software and Algorithms		
pClamp10 software	Molecular Devices	
CRISPR Design	Zhang Lab, MIT	http://crispr.mit.edu/
PhenoMaster	TSE	https://www.tse-systems.com/product-details/phenomaster
MATLAB	Mathworks	https://www.mathworks.com/
Prism6	GraphPad	https://www.graphpad.com/scientific-software/prism/

CONTACT FOR REAGENT AND RESOURCE SHARING

Further information and requests for reagents may be directed to the Lead Contact Lora Heisler (lora.heisler@abdn.ac.uk).

EXPERIMENTAL MODEL AND SUBJECT DETAILS

Mice

5-HT$_{2C}$R-ires^{Cre} (Burke et al., 2016; Marcinkiewcz et al., 2016), *loxtb5-HT$_{2C}$R* (B6.129-*Htr2c*^{tm1Jke}/J; The Jackson Laboratory, Bar Harbor, ME USA), *Pomc*^{DsRED} (Hentges et al., 2009) and *Pomc^{NEO}* (Bumaschny et al., 2012) mice were housed with *ad libitum* food and water access (unless otherwise stated) in a light- (12 h on/12 h off) and temperature-controlled (21.5°C to 22.5°C) environment. All procedures were performed in accordance with the U.K. Animals (Scientific Procedures) Act 1986 and local ethical approvals.

METHOD DETAILS

Stereotaxic Surgery and Viral Vectors

Viral constructs were packaged in AAV serotype-8 and were delivered to the NTS via bilateral stereotaxic injections, as previously described (D'Agostino et al., 2016).

AAV vectors

The DREADD viruses used have been described previously: AAV8-hSyn-DIO-hM3D$_q$-mCherry, AAV8-hSyn-DIO-hM4D$_i$-mCherry and AAV8-hSyn-mCherry-Cre were packaged in AAV serotype-8 at a titer of 1.3 x 10^{13} vg/ml (University North Carolina Vector Core, Chapel Hill, NC, USA) (Krashes et al., 2011; D'Agostino et al., 2016). AAV/DJ-CMV-spCas9 was obtained from Vector Biolabs (Cat No. 7120; Vector Biolabs, Malvern, PA, USA). High titer pAAV-U6sgRNA(SapI)_hSyn-GFP-KASH-bGH (PX552) were packaged in AAV serotype-8 at a titer of 4.1 x 10^{12} vg/ml (Unitat de Producció de Vectors, Universitat Autonoma Barcelona, Spain).

Stereotaxic Surgery

NTS delivery of AAVs was achieved through a stereotaxic procedure. Briefly, 5–8 week old male mice were anesthetized with a mixture of ketamine and xylazine dissolved in saline (100 and 10 mg/kg, respectively injected in a volume of 10 ml/kg, i.p.). Mice were placed in a stereotaxic frame (Kopf Instruments, Tujunga, CA USA) using ear bars with the head angled at 45°. Under magnification, an incision was made at the level of the cisterna magna and neck muscles carefully retracted. A 33G needle was used for dura incision. The obex served as reference point for injection. Bilateral injections were performed using a glass micropipette (diameter 20–40 μm). NTS coordinates used were A/P, -0.2; M/L, ±0.2; D/V, -0.2 from the obex. Virus was delivered under air pressure using a PLI-100A Pico-Injector (Harvard Apparatus, Cambridge, UK). 150 nl of virus per side was delivered with multiple

microinjections over 4–5 minutes. The pipette remained in place for a minimum of 3 minutes after injection. Following all surgery, animals were closely monitored, kept warm and appropriate postsurgical care was taken. Animals were also administered an analgesic (5 mg/kg carprofen, s.c.) daily for 2 days post-operatively. Prior to experimentation, mice were acclimated to handling and i.p. injections.

Mice were given a minimum of 14 days of surgical recovery before experimentation. For chemogenetic experiments, mCherry was visualised by immunohistochemistry. Mice in which the expression of reporter fluorescent protein was bilaterally detected in the caudal aspect of the DVC were included in the analysis, whereas mice without reporter fluorescent expression or expression outside the DVC were excluded from the analysis. Caudal DVC was defined by the presence of the area postrema in the same section of brain tissue.

DNA Constructs

For SpCas9 target selection and generation of single guide RNA (sgRNA), the 20-nt target sequences were selected to precede a 5'-NGG protospacer-adjacent motif (PAM) sequence. To minimize off-targeting effects, the CRISPR design tool was used (http://crispr.mit.edu/). The 20-nt target and complement sequences were synthesized to include BbsI overhangs. Oligos were annealed, phosphorylated and ligated into the pX330-U6-Chimeric_BB-CBh-hSpCas9 (PX330; Addgene plasmid #42230) plasmid after BbsI digestion.

pAAV-U6sgRNA(SapI)_hSyn-GFP-KASH-bGH (PX552; Addgene plasmid # 60958) was used for cloning sgRNAs into AAV the backbone and generate viral particles. The plasmid was first digested with SapI to create sticky ends for ligation. The 20-nt target and complement sequences were synthesized to include SapI overhangs and ligated. A second sgRNA was then inserted into this vector. The second sgRNA was first PCR-amplified from PX330. Primers were designed to include 15bp homology arms to the PspOMI-lineraised PX552 into the PCR clone sgRNA guide. The PCR product was column-purified and then cloned into the PspOMI-lineraised PX552 vector via homologous recombination-assisted cloning (In-Fusion HD cloning Kit®, Cat No. 638910; Clonotech Laboratories). Primers sequences: AGACTGCAGAGGGCCGAGGGCCTATTTCCCATGATTCCT, forward; CTCATACGCAGGGCCCTAAAACAAAAAAGCACCGACTCGG, reverse. All obtained constructs were verified by sequencing. All plasmids were amplified using One Shot® Stbl3™ Chemically Competent *E. coli* (Cat No. C737303; Invitrogen) and column-purified (Cat No. 270104; Qiagen).

Pomc targeting sgRNAs (5' to 3'): sgRNA 1- GGTGGGCAAGAAACGGCGCC(cgg); sgRNA 2- GTGACCCATGACGTACTTCCG(ggg).

Cell Line Culture and Transfection

Mouse Neuro-2a (N2a) cells were grown in DMEM containing 10% FBS. Cells were maintained at 37°C in 5% CO_2 atmosphere. Transfection were performed using FuGENE ® HD Transfection Reagent (Cat No E2311; Promega) according to the manufacturer's protocol. Briefly, $1.5x10^4$ cells per well were plated into a 12 well plate the day before transfection. 1 ug of PX330 plasmid was mixed with Fugene HD in Optimem medium (ratio 1:3). Cells were incubated for 48 hours. Genomic DNA was extracted from transfected cells using PureLink® Genomic DNA Mini Kit (Cat No K182001, Invitrogen) following the manufacturer's instructions.

Detection of Insertions/Deletions (Indels)

The third exon of the mouse *Pomc* gene was PCR amplified from genomic DNA using the following primers: GCTTGCATCCGGGCTTGC, *forward*; GACTTTATTTACGCAGTTTTTATTGAAGATCAGAGC, *reverse*. This PCR reaction generates a single PCR product of the expected 726bp size. PCR product was sequence verified. To detect sgRNA/spCas9-induced *indels*, genomic DNA from cells was amplified using the same primers and this 726bp product corresponding to third exon of the mouse *Pomc* gene was used for the endonuclease I assay (Surveyor® Mutation Detection Kit; Cat No. 706020; Integrated DNA Technologies) following the manufacturer's instructions. Briefly, PCR amplicons were heat-denaturated and re-annealed using a thermocycler to create DNA heteroduplexes. DNA heteroduplexes were incubated with endonuclease I enzyme, which cleaves DNA at the site of a base mismatch with high specificity, for 1 hour at 42°C. Genomic modifications were verified using gel agarose electrophoresis.

Feeding Studies

Dark Cycle Food Intake

Mice were injected with vehicle or drug 30 minutes prior to the onset of the dark cycle and food was removed. At the onset of dark, food was returned and manually (home cage) or automatically (Phenomaster chambers, TSE, Bad Homburg, Germany) weighed using methods previously described (D'Agostino et al., 2016).

Post-fast Refeeding

Food was removed at the onset of the dark cycle and returned 2 hours after the onset of the light cycle the following day. Food intake was measured manually (home cage) or automatically (TSE Phenomaster chambers) using methods previously described (D'Agostino et al., 2016).

Progressive Ratio

Mice were trained in operant chambers housed in sound-attenuating boxes and controlled by a PC Med-PC-IV programming language (MED Associates, Inc., Fairfax, VT USA). Chambers (21.6 cm long × 17.8 cm wide × 12.7 cm high) have a retractable lever, a pellet receptacle and a 3 watt house-light on the opposite wall (Cat No. MED-307W-D1, MED Associates, Inc.). Under food restriction, mice were trained to press the lever for 20 mg chocolate pellet reinforcers (TestDiet, St. Louis, MO USA) under a fixed ratio (FR) 1 schedule for 3 days followed by FR 5 for a further 3 days. Then they were fed *ad libitum* and underwent daily training under a PR schedule based on an exponential progression derived from the formula (5 × e0.2n)−5, rounded to the nearest integer,

where n is the position in the ratio sequence (Richardson and Roberts, 1996). 50 minute sessions took place at the same time each day during the light phase (between 08:00 and 13:00 hours) for 1 week. The breakpoint or alternatively the highest completed ratio (Olarte-Sánchez et al., 2012) was defined as the last ratio completed before 5 minutes elapsed without any responding.

Immunohistochemistry (IHC) and Fluorescent In Situ Hybridization Histochemistry (FISH)

IHC. Tissue was processed for GFP, mCherry, dsRed and/or c-Fos (FOS-IR) IHC as previously described (D'Agostino et al., 2016). Briefly, mice were transcardially perfused with phosphate buffered saline (PBS) followed by 10% neutral buffered formalin (Sigma-Aldrich). Brains were extracted, post-fixed in 10% neutral buffered formalin at 4°C, cryoprotected in 20% sucrose at 4°C and then sectioned coronally on a freezing sliding microtome at 25 μm. Tissue was processed for chicken anti-GFP (1:1000; AbCam, ab13970), rabbit anti-dsRED (1:1000; Rockland, 600-401-379), goat anti-mCherry/RFP (1:1000; Sicgen, AB0040-200) or anti-c-FOS (1:5000, rabbit, Calbiochem, PC38) primary antibodies and a biotinylated donkey anti-rabbit (1:500, Jackson ImmunoResearch Laboratories, Inc.) or Alexa Fluor (1:500, Life Technologies) secondary antibodies using standard protocols previously described (Lam et al., 2009; Heisler et al., 2006). Tissue was then mounted on slides, cover slipped and the NTS visualized using an Axioskop II microscope (Carl Zeiss, Oberkochen, Germany) and Adobe Photoshop CS5 software. Images of single-label immuoreactivity (IR) for GFP or mCherry were used to visualize and analyze NTS injection sites.

Dual-IHC

Dual-IHC was performed to visualize co-expression of mCherry-IR and FOS-IR in *5-HT$_{2C}$R*^{NTS}-hM3D$_q$ mice. Specifically, mice were fasted overnight and then injected with designer drug CNO or saline. Brains were extracted 90 minutes later and dual-label mCherry and FOS-IR performed using method outlined above. Quantitative analysis of single and dual-labelled cells was performed manually using an Axioskop II microscope and Adobe Photoshop CS5 software.

IHC and FISH

IHC and FISH were performed to visualize co-expression of POMC and 5-HT$_{2C}$R within the NTS in *POMC^{dsRED}* mice. To label *5-HT$_{2C}$R* expressing neurons, a RNA expression vector (pBluescript SK-) containing the 3-kilobase (kb) coding region of 5-HT$_{2C}$R cDNA was used to generate single-stranded RNA probes (Julius et al., 1988). Briefly, *in vitro* transcription was performed using Digoxigenin (DIG)-RNA Labelling Mix (Roche, Mannheim, Germany). Sections were treated with 1% Sodium Borohydride solution and 0.25% Acetic Anhydride in Triethanolamine (TEA) solution. Sections were then incubated in a hybridization buffer containing a DIG-UTP-labelled 5-HT$_{2C}$R riboprobe overnight at 55°C. Sections were rinsed in a standard sodium citrate/50% formamide solution, rinsed in an RNase (0.02 mg/ml RNase A) solution, incubated in a 3% H_2O_2 solution for 30 minutes and blocked in a 2% sheep serum (Sigma-Aldrich, Saint Louis, MO USA). Sections were then incubated with an anti-DIG antibody (1:5000, Roche, Mannheim, Germany), treated with TSA PLUS Biotin Kit (PerkinElmer Inc., Waltham, MA USA) and revealed with streptavidin conjugated Alexa Fluor® 568 (1:2000, Life Technologies, Carlsbad, CA USA). To label POMC, sections were blocked again and incubated with rabbit anti-dsRED (1:1000; Rockland, Limerick, PA USA; 600-401-379) overnight at 4°C using the IHC protocol described above. The sections were incubated with Alexa Fluor® 568 donkey anti-rabbit secondary antibody (1:500, Invitrogen™ Life Technologies, Carlsbad, CA USA) for 1 hour. Tissue was then mounted on slides, cover slipped and single- and dual-labelling assessed in the NTS using an Axioskop II microscope and Adobe Photoshop CS5 software. Quantitative analysis of single and dual-labelled dsRed-IR and *5-HT$_{2C}$R* mRNA cells was conducted manually.

Enzyme Immunoassay (EIA) for Alpha-Melanocyte-Stimulating Hormone (Alpha-MSH)

Mice were anesthetized with sodium pentobarbital (Euthatal) and decapitated. The brain was rapidly removed, the hypothalamus was dissected using razor blades and mouse acrylic brain matrices (Stoelting Co.) and frozen in dry ice. Hypothalamic lysates were obtained using a probe sonicator. Lysis consisted of an acid-ethanol solution obtained by combining 1 part of concentrated HCl with 7 parts pure ethanol. 0.1ml of lysis was used per 10 mg of tissue (weight of each hypothalamic explant was about 30 mg). 10 μl of crude homogenate were combined with 50 μl of fresh lysis buffer and centrifuged 3500 g for 30 min. Supernatants were collected and dried using a vacuum centrifuge in low protein binding tubes (Eppendorf® LoBind, Sigma-Aldrich, Cat No. Z666505). Dry fractions were solubilised in 150 μl of assay buffer and alpha-MSH content analysed using a fluorescent EIA ultra-sensitive assay (Phoenix Pharmaceutical Inc.; Cat No FEK-043-01) following the manufacturer's instructions.

Electrophysiology and Chemogenetic Validation Analysis

For electrophysiological experiments, male and female *POMC$^{DS\text{-}Red}$* (n=44), male *loxtb5-HT$_{2C}$R* (n=9), male *5-HT$_{2C}$R*^{NTS}-hM3D$_q$ (n=2) or male *5-HT$_{2C}$R*^{NTS}-hM4D$_i$ (n=2) mice aged 2-6 months were anesthetized with sodium pentobarbital (Euthatal) and decapitated. The brain was rapidly removed and placed in cold (0–4°C), oxygenated (95%O_2/5%CO_2) 'slicing' solution containing (in mM) sucrose (214), KCl (2.5), NaH_2PO_4 (1.2), $NaHCO_3$ (26), $MgSO_4$ (4), $CaCl_2$ (0.1), D-glucose (10). The brain was fixed to a vibrating microtome (Campden Instruments, Loughborough, UK) and 200-μm thick coronal sections of the brainstem containing the NTS were prepared. Slices were immediately transferred to a 'recording' solution containing (in mM) NaCl (127), KCl (2.5), NaH_2PO_4 (1.2), $NaHCO_3$ (26), $MgCl_2$ (1.3), $CaCl_2$ (2.4) and D-glucose (10) in a continuously oxygenated holding chamber at 35°C for a period of 25 minutes. Slices were remained in the recording solution at room temperature for a minimum of 1 hour before recording. For whole-cell recordings, slices were transferred to a submerged chamber and a Slicescope upright microscope (Scientifica, Uckfield, UK) was used for infrared - differential interference contrast and fluorescence visualization of cells. During recording, slices were continuously perfused at a rate of ca. 2 ml/minute with oxygenated recording solution at room temperature. All pharmacological compounds were bath applied. Whole cell current-clamp recordings were performed with pipettes (3–7 MΩ when filled with intracellular solution)

made from borosilicate glass capillaries (World Precision Instruments, Aston, UK) pulled on a Zeitz DMZ micropipette puller (Zeitz Instruments GmBH, Martinsried, Germany). The intracellular recording solution contained (in mM) K-gluconate (140), KCl (10), HEPES (10), EGTA (1), Na_2ATP (2), pH 7.3 (with KOH). Recordings were performed using a Multiclamp 700B amplifier and pClamp10 software (Molecular Devices, Sunnyvale, CA USA). Liquid junction potential was 16.4 mV and not compensated. The recorded current was sampled at 10 kHz and filtered at 2 kHz unless otherwise stated.

Drugs

Lorcaserin (LGM Pharma, Erlanger KY USA), WAY161,503 (Tocris Bioscience, Abingdon UK) and clozapine-n-oxide (CNO; Tocris Bioscience) were prepared in double distilled H_2O, diluted in sterile phosphate-buffered saline (PBS) and administered *in vivo* i.p. in a volume of 10 ml/kg. 5-HT (Sigma-Aldrich), lorcaserin, WAY161,503 and CNO were dissolved in artificial cerebrospinal fluid (aCSF) for electrophysiology experiments.

QUANTIFICATION AND STATISTICAL ANALYSIS

Data were analyzed with t-test, Mann Whitney U test, One-way, Two-way or Repeated Measures ANOVA followed by Tukey's or Sidak's *post hoc* tests, where appropriate. For all analyses, significance was assigned at $p<0.05$. Data are presented as mean±SEM. All statistical analyses were completed with GraphPad Prism 7.0 (GraphPad Software, San Diego, CA USA).

Cell Metabolism, Volume *28*

Supplemental Information

Nucleus of the Solitary Tract

Serotonin 5-HT_{2C} Receptors Modulate Food Intake

Giuseppe D'Agostino, David Lyons, Claudia Cristiano, Miriam Lettieri, Cristian Olarte-Sanchez, Luke K. Burke, Megan Greenwald-Yarnell, Celine Cansell, Barbora Doslikova, Teodora Georgescu, Pablo Blanco Martinez de Morentin, Martin G. Myers Jr., Justin J. Rochford, and Lora K. Heisler

Supplemental Figures

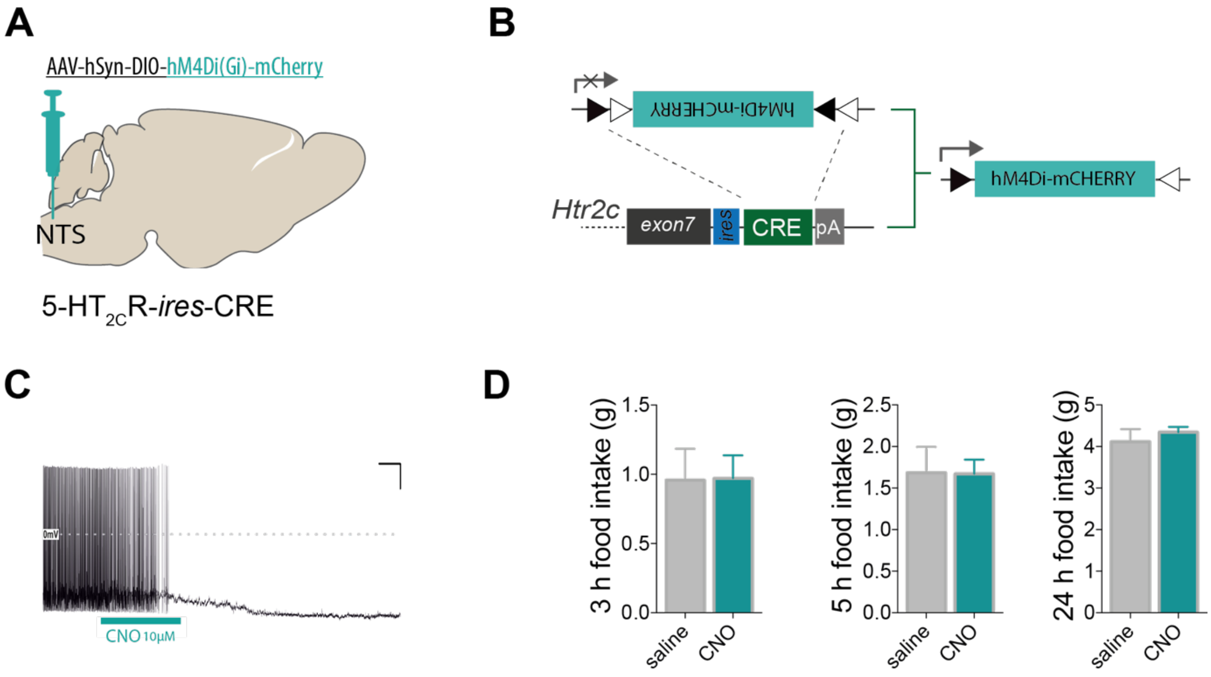

Figure S1, related to Figure 1. (A) Schematic of the strategy used to inhibit the activity of 5-HT_{2C}R-expressing neurons within the NTS: *5-HT$_{2C}$R^{CRE}* mice were stereotaxically injected into the NTS with a Cre-dependent DREADD vector (AAV-hM4D_i-mCherry). (**B)** *5-HT$_{2C}$R^{CRE}* construct and Cre-mediated recombination of DREADD allele within the NTS produced *5-HT$_{2C}$R*^{NTS}-hM4D_i mice. **(C)** Representative example of electrophysiological recording of an isolated hDM4i-transduced 5-HT_{2C}R-expressing neuron from the NTS of *5-HT$_{2C}$R*^{NTS}-hM4D_i mice illustrating that CNO (10 µM) caused a reduction in the membrane potential (n=5/5). **(D)** Chemogenetic inhibition of 5-$HT_{2C}R^{NTS}$ neurons via CNO (1 mg/kg, i.p.) administration in *5-HT$_{2C}$R*^{NTS}-hM4D_i mice did not alter food intake compared to saline up to 24 hours post-treatment (n= 6-7; 3h: $t(11)=0.044$, $p=0.965$; 5h: $t(11)=0.038$, $p=0.970$; 24h: $t(11)=0.642$, $p=0.533$). Data are presented as mean±SEM.

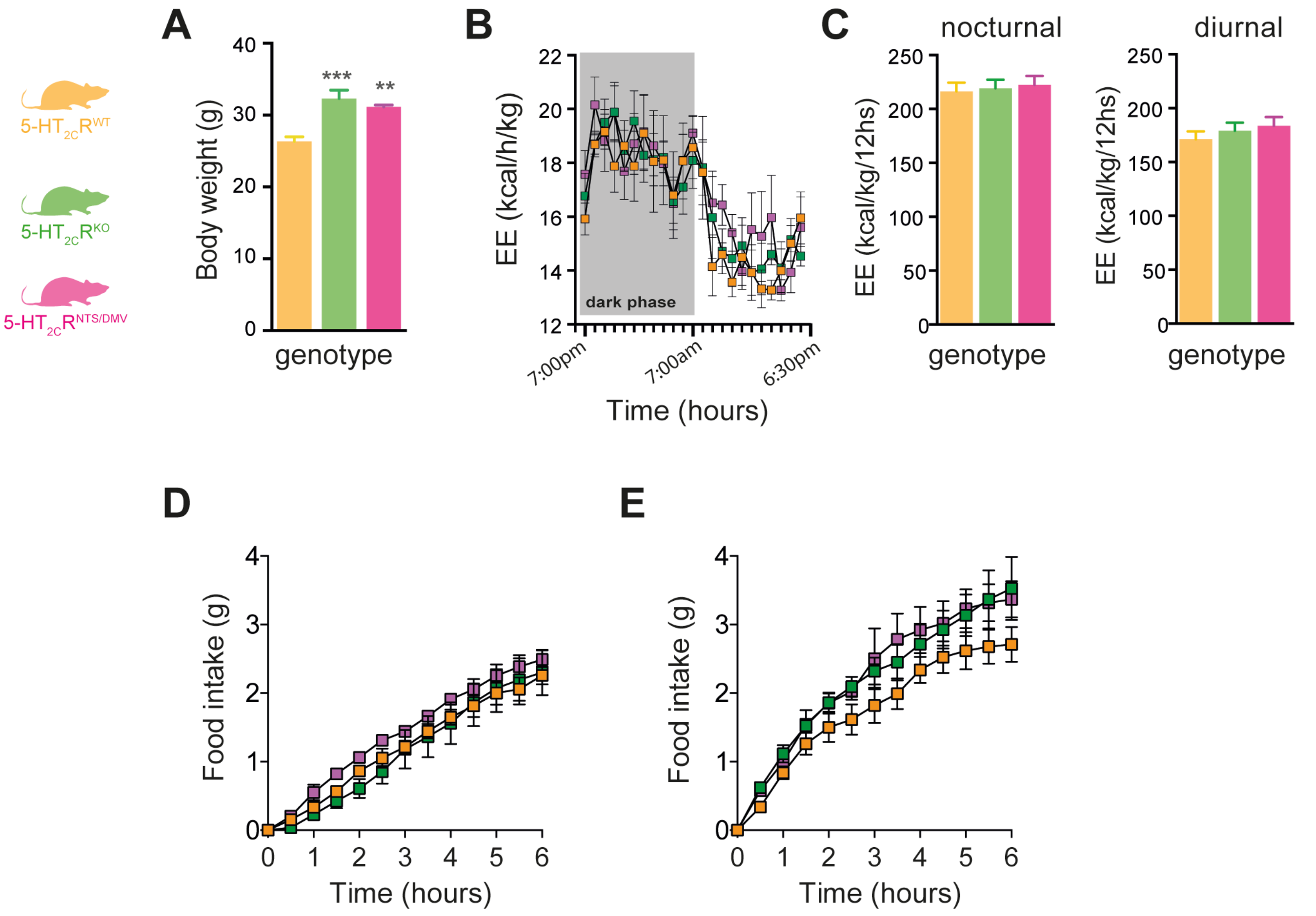

Figure S2, related to Figure 2. (A) Body weight of 4 month old male wild type (*5-$HT_{2C}R^{WT}$*), *loxtb5-$HT_{2C}R$* null mice (*5-$HT_{2C}R^{KO}$*) and *loxtb5-$HT_{2C}R$* null mice with 5-HT_{2C}Rs exclusively restored within the NTS/DMV following stereotaxic delivery of AAV-hSyn-Cre-mCherry into the NTS/DMV (*5-$HT_{2C}R^{NTS/DMV}$*) (n=5-6 per group, $F_{2,16}$= 14.40, p=0.0003). **(B)** Representative recording of circadian energy expenditure (EE) measured by indirect calorimetry illustrating no effect of genotype on EE (kcal/h/kg) during the dark or light phase of the daily cycle in *5-$HT_{2C}R^{WT}$* (n=5), *5-$HT_{2C}R^{KO}$* (n=5) or *5-$HT_{2C}R^{NTS/DMV}$* (n=6) mice **(**Effect of genotype $F_{2,13}$ = 0.26, p = 0.7739; effect of time $F_{23,299}$ = 15.56, p <0.001; interaction $F_{46,299}$ = 0.66, p = 0.9544); **(C)** Data expressed as cumulative nocturnal and diurnal EE also illustrate no differences by genotype in EE. **(D)** Cumulative *ad libitum* dark cycle food intake illustrating no differences by genotype. **(E)** Cumulative light cycle food intake after an overnight fast illustrating no significant differences by genotype. Sidak's post hoc comparisons **p < 0.01, ***p < 0.001 compared to *5-$HT_{2C}R^{WT}$* mice. Data are presented as mean±SEM.
